# Amino acids integrate behaviors in nerveless placozoans

**DOI:** 10.3389/fnins.2023.1125624

**Published:** 2023-04-13

**Authors:** Mikhail A. Nikitin, Daria Y. Romanova, Simkha I. Borman, Leonid L. Moroz

**Affiliations:** ^1^Belozersky Institute of Physico-Chemical Biology, Lomonosov Moscow State University, Moscow, Russia; ^2^Institute of Higher Nervous Activity and Neurophysiology of RAS, Moscow, Russia; ^3^Koltzov Institute of Developmental Biology Russian Academy of Sciences, Moscow, Russia; ^4^Departments of Neuroscience and McKnight Brain Institute, University of Florida, Gainesville, FL, United States; ^5^Whitney Laboratory for Marine Bioscience, University of Florida, St. Augustine, FL, United States

**Keywords:** Placozoa, glutamate, GABA, ATP, evolution of nervous system, feeding, glycine, locomotion

## Abstract

Placozoans are the simplest known free-living animals without recognized neurons and muscles but a complex behavioral repertoire. However, mechanisms and cellular bases of behavioral coordination are unknown. Here, using *Trichoplax adhaerens* as a model, we described 0.02–0.002 Hz oscillations in locomotory and feeding patterns as evidence of complex multicellular integration; and showed their dependence on the endogenous secretion of signal molecules. Evolutionary conserved low-molecular-weight transmitters (glutamate, aspartate, glycine, GABA, and ATP) acted as coordinators of distinct locomotory and feeding patterns. Specifically, L-glutamate induced and partially mimicked endogenous feeding cycles, whereas glycine and GABA suppressed feeding. ATP-modified feeding is complex, first causing feeding-like cycles and then suppressing feeding. *Trichoplax* locomotion was modulated by glycine, GABA, and, surprisingly, by animals’ own mucus trails. Mucus triples locomotory speed compared to clean substrates. Glycine and GABA increased the frequency of turns. The effects of the amino acids are likely mediated by numerous receptors (R), including those from ionotropic GluRs, metabotropic GluRs, and GABA-BR families. Eighty-five of these receptors are encoded in the *Trichoplax* genome, more than in any other animal sequenced. Phylogenetic reconstructions illuminate massive lineage-specific expansions of amino acid receptors in Placozoa, Cnidaria, and Porifera and parallel evolution of nutritional sensing. Furthermore, we view the integration of feeding behaviors in nerveless animals by amino acids as ancestral exaptations that pave the way for co-options of glutamate, glycine, GABA, and ATP as classical neurotransmitters in eumetazoans.

## Introduction

The nervous systems’ origins are among the major evolutionary transitions in the history of life. During such transitions, the canonical, synaptically-wired nervous systems could be preceded by the so-called “volume transmission type integrative systems,” composed of heterogeneous populations of secretory cells releasing signal molecules (Moroz, 2009, 2021). Volume transmission might lack speed and spatial localization of synaptic transmission, but in the Proterozoic world, without macroscopic predators, it could be sufficient for coordinating behaviors in early animals. The overall hypothesis is that signaling molecules, initially acquired for volume transmission systems, later in evolution were co-opted as neurotransmitters and hormones (Moroz et al., 2021b). What are these signaling molecules, and how do they control behaviors in simplest animals?

Sponges (Porifera) and placozoans (Placozoa) are the two extant early-branching animal lineages, primarily lacking neurons, chemical, and electrical synapses but capable of complex and coordinated behaviors with about a dozen of cell types (Smith et al., 2014; Musser et al., 2021). Thus, both phyla are critical in deciphering mechanisms of neural evolution (Smith et al., 2015; Senatore et al., 2017; Moroz, 2018; Varoqueaux et al., 2018; Moroz and Romanova, 2021, 2022). Sponges are sedentary filtrators, while placozoans are highly motile, feed on macroscopic objects (bacterial and algal biofilms), and display various motor complexes, including exploratory and social behaviors (Okshtein, 1987; Seravin, 1989; Smith et al., 2015; Senatore et al., 2017; Armon et al., 2018; Fortunato and Aktipis, 2019; Smith et al., 2019; Romanova et al., 2020a).

Therefore, we used *Trichoplax adhaerens* as the primary reference species to analyze the cellular bases of placozoan behaviors and focused on identifying individual intercellular signal molecules. The 3-layer cellular organization in Placozoa features functional asymmetry, underlying observed behavioral outcomes. The lower epithelium acts as the densely ciliated locomotory surface with a vertical orientation of digestive/secretory cells supporting feeding. In contrast, the upper contractive layer is more flattened and performs a defensive function against predators. The middle layer consists of more horizontally oriented so-called fiber cells with supposed integrative functions (Grell and Ruthmann, 1991; Smith et al., 2014; Romanova et al., 2021). Fiber cells and, perhaps, other smaller cells in the area form a meshwork of neural-like cell processes containing structures resembling secretory sites or exosomes (Hoshino et al., 2013; Smith et al., 2014; Romanova et al., 2021, 2022) Furthermore, microcavities between ventral and middle cell layers might contribute to the adsorption and sensing of metabolites (Moroz et al., 2021b).

Spaces between ventral epithelium and fiber cells might also contribute to the formation of a compartmentalized dynamic chemical microenvironment with a mixture of signal molecules, which can be both products of enzymatic digestion and specifically secreted molecules. Within this intercellular space, internally and locally secreted signal molecules could access different cellular targets supporting the integration of behaviors (Moroz et al., 2021b).

Thus, in the absence of canonical neurons and muscles, such simpler cellular architecture could facilitate volume transmission to be sufficient to coordinate local contractions, rapid ciliary beating, and even complex behaviors without synapses. However, the nature of signal molecules in Placozoa remains uncertain. In addition to several small peptides (Nikitin, 2015; Varoqueaux et al., 2018) low molecular weight transmitters such as nitric oxide (NO; Moroz et al., 2020a) and glycine (Romanova et al., 2020a) were implicated in coordinating behaviors.

Here, we test whether various amino acids (also acting as digestion products and more localized cell-specific release molecules) can control and integrate placozoan locomotion and feeding behaviors. Due to limited ethological observations, we first described *Trichoplax* behaviors with and without food substrates. Next, we characterized the action of glutamate and aspartate enantiomers, GABA, and glycine as one of the most abundant metabolites in *Trichoplax* (Moroz et al., 2020b). Finally, we tested the effects of ATP – the evolutionary ancient and critical component of synaptic/exocytosis vesicles, and purinergic transmission in general. The data indicate that *Trichoplax* can sense amino acids and suggest that different amino acids integrate behaviors in nerveless placozoans.

## Materials and methods

### Long-term culturing of placozoans

We used clonal cultures of *Trichoplax adhaerens* (Grell’s strain Н1, from the Red Sea). We maintained individuals in closed Petri dishes with artificial seawater (ASW, 35 ppm, pH 3–5 days), which was changed (70% of the total volume) every 3–5 days. At the same time, a suspension of the green alga *Tetraselmis marina* (WoRMS Aphia, ID 376158) was added to the culture dishes. We maintain placozoans at the constant temperature of 24 ^0^С and natural light in environmental chambers (Romanova et al., 2022).

### Behavioral experiments

**Figure 1:**
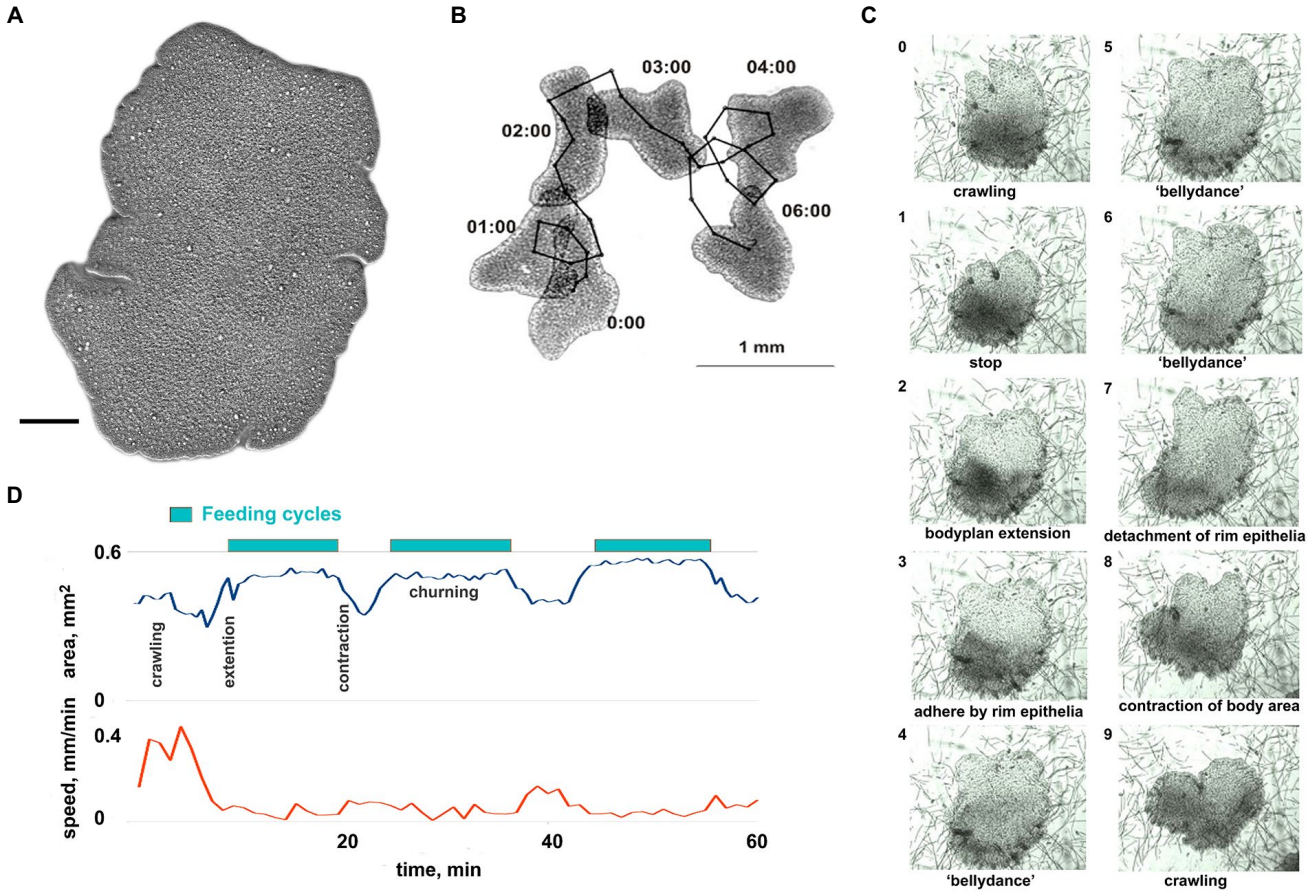
General overview of *Trichoplax* behavior. **(A)** *Trichoplax adhaerens* (photo) is a simple, irregularly shaped flat animal with three cell layers. Muscles, neurons, gastric cavities, or any organs are absent. Scale bar = 0.1 mm. **(B)** Example of *Trichoplax* movement and tracking. Superimposed images of an animal every 60 s are shown. Black dots mark the positions of animal centroid every 10 s. **(C)** The sequence of ten images shows the feeding cycle of *Trichoplax* on the cyanobacterial biofilm. Images were captured every minute. The animal ceases movement and expands at the beginning of each feeding cycle. The body edge is attached to a substrate, enzymes are secreted, and digested food is absorbed. Digestion is enhanced by churning movements of the central part of the animal. After a digested food is absorbed, the animal detaches its edges, contracts, and resumes movement. Each cycle takes approx. 10–20 min. **(D)** The plot of the body area and crawling speed (mm/min) for representative animal feeding cycles on cyanobacterial biofilm. Body area increases at the beginning of each feeding cycle and decreases at the end of each cycle. Locomotion is suppressed during each feeding cycle.

We have routinely observed the relatively stereotyped feeding cycles with the green algae *Tetraselmis* or cyanobacteria *Oscillatoria* as food (Figure 1C).

However, on cyanobacteria *Oscillatoria,* animals can unexpectedly stop feeding and begin to glide around for several hours (Supplementary Figure S2). We could not reproduce these long gliding episodes or elucidate their factors. Therefore, only *Tetraselmis* biofilm has been used in pharmacological tests on feeding behavior (below). Nevertheless, we occasionally employed cyanobacterial biofilm for illustrative purposes of stereotyped patterns (Supplementary Videos S2, S9, S10) because of its uniform color and absence of carbonate sediments.

We have recorded 861 h of time-lapse video, usually recording 8–12 animals at once (over 8,000 animal hours). This dataset included 280 hours (h) of normal behavior (118 h on clean glass and 162 h on algal biofilms), 32 h of experiments with altered salt composition, 96 h with glutamate and aspartate, 278 h with GABA, 75 h with glycine, and 100 h with ATP.

Animal behavior was observed in small (52 mm) glass Petri dishes. Canon PowerShot SX100 camera in macro mode and Gphoto2 software was used for making time-lapse photo series of entire Petri dish with 10 or 30 s intervals. To increase contrast, the experimental Petri dish was installed on a wet black background and illuminated by a thin (3 mm) layer of light from all sides using a custom-made illumination device.

Experimental animals were transferred from a cultural dish in the late exponential phase to a small clean Petri dish for 1 h. They transferred to a glass Petri dish for behavior ecording. 8–12 animals were filmed simultaneously in each experiment.

Experimental glass Petri dishes with algal biofilms were prepared for some experiments. Growing an algal biofilm from small inoculate invariably introduces numerous bright calcareous inclusions which interfere with time-lapse recording and video processing. For cyanobacteria, we placed 5–6 pieces of *Oscillatoria* biofilm (size ~ 1 mm) from cultural to experimental dish with clean ASW, waited 2–3 days for cyanobacterial filaments to spread across the dish, and gently removed residual pieces of transferred thick biofilm. After another 24 h, cyanobacteria spread to clean patches left after removing dense biofilm remnants, and fresh, uniformly thin biofilm was ready for experiments. For green algae biofilm, we scraped some *Tetraselmis* cells from a cultural dish with a pipette, transferred 200–300 μL of the cell suspension to a microcentrifuge tube, and waited for 1 min. After that, we gently moved most of the suspension (discarding the bottom 50 μL with calcareous sediment) to the experimental dish and waited for 1–2 h for algal cells to attach to the glass. All amino acids and ATP were from Sigma-Aldrich. Stock solutions were prepared for each experimental day.

Ca-free and high-Mg artificial seawater (ASW) recipes were based on the ASW recipe from Cold Springs Harbor Protocols (Seawater, 2012). Ca-free artificial seawater was prepared as follows: NaCl: 436 mM; NaHCO_3_: 2 mM; KCl: 9 mM; MgCl_2_x6H_2_O: 23 mM; MgSO_4_x7H_2_O: 25.5 mM; dissolved in distilled water. For behavior experiments with low Ca^2+,^ animals were initially recorded in ~1 ml drops of normal ASW (10 mM CaCl_2_) and then 6X volume of Ca-free seawater was added to the final concentration of CaCl_2_ 1.5 mM.

For behavior experiments with 200 mM MgCl_2_ animals were initially recorded in normal ASW (48 mM MgCl_2_) and then an equal volume of high-Mg seawater was added to a final concentration of MgCl_2_ 200 mM. We have tested the effects of ASW with 200 mM MgCl_2_ and appropriately decreased Na^+^ (standard ASW contains 48 mM MgCl_2_).

### Analysis of behavior recordings

Time-lapse sequences of *Trichoplax* behavior were imported to the Fiji ImageJ program (Schindelin et al., 2012). Animal positions and paths were determined using the WrMtrck plugin (Nussbaum-Krammer et al., 2015). Illustrative videos were created from the same time-lapse photo series using Avidemux video editor^1^.

Track data was analyzed in OpenOffice Calc (graphs, Welch *t*-test, straightness and turn angle calculation). There are several measures of animal path tortuosity described in the literature: Intensity of Habitat use (IU), Fractal D, MSD (Mean Squared Distance), Straightness (ST), and Sinuosity (SI; Benhamou, 2004; Almeida et al., 2010). In our data, path shape changes in different conditions were best captured by the simplest of these measures, the straightness index, which is just displacement (linear distance between start and finish) divided by path length. It takes values from 0 (closed loop) to 1 (straight path). We used 5-min and 60-min straightness in this work.

Feeding cycles were counted manually. Power spectral density was calculated using the Signal package from SciPy library (Virtanen et al., 2020) using a custom Python script.

### Search and annotation of iGluR genes, alignment, and phylogenetic trees

We used the data from the sequenced genomes of animals deposited in GenBank and elsewhere (listed in Supplementary Table S2). The search for possible homologs was performed using sequence similarity methods (BLAST/DELTA BLAST) algorithm using all human iGluRs, mGluRs, GABA-Brs, and vGluTs from SwissProt database as initial queries. Protein sequences were aligned in MAFFT Online (Katoh et al., 2005, 2019).^2^ Phylogenetic trees were inferred using either the Maximum Likelihood algorithm implemented in IQTREE 1.6.12 (Nguyen et al., 2015) with automatic choice of evolutionary models or the Bayesian algorithm in MrBayes 3.2.6 (Ronquist et al., 2012). Maximum Likelihood tree robustness was tested with 2000 ultrafast bootstrap replicates (Hoang et al., 2018). Trees were visualized and annotated in iTol WEB (Letunic and Bork, 2021).

## Results

### Native behavior of *Trichoplax adhaerens*

#### Search and feeding cycles as dominant behavioral patterns

*Trichoplax* behavior is substantially affected by substrate composition and food abundance, with relatively stereotyped feeding episodes. Without food, *Trichoplax* glides on ventral cilia without defined anterior end and constantly changing shape (Figures 1A,B; Supplementary Video S1). Its path is apparently chaotic and reminiscent of Brownian motion (Figure 1B). On suitable algal (*Tetraselmis*) or cyanobacteria (*Oscillatoria*) substrates, animals could move as little as 0.05 mm before the next feeding cycle, which is often initiated upon contact with microalgae (Smith et al., 2015; Senatore et al., 2017; Smith et al., 2019; Smith and Mayorova, 2019).

Each cycle takes approximately 10–20 min and begins with a locomotory pause, also associated with expanding the body surface area (Figures 1C,D; Supplementary Video S2). *Trichoplax* adhere to the substrate, secrete digestive enzymes on the surface below, and perform “churning” or swirl-like movements of the central area with body edges tightly attached. *Trichoplax* absorbs the contents of lysed algal cells during this period. After the 5–15 min absorption phase, animals detach their peripheral edges, decrease body surface area, and resume locomotion.

Long-term experiments show that feeding cycles on *Tetraselmis* biofilm are robust, stereotyped, and reproducible for 24–30 h. Therefore, *Tetraselmis* biofilm has been used in pharmacological tests on feeding and exploratory behavior (see below).

#### Low-frequency oscillations of *Trichoplax* behaviors

**Figure 2:**
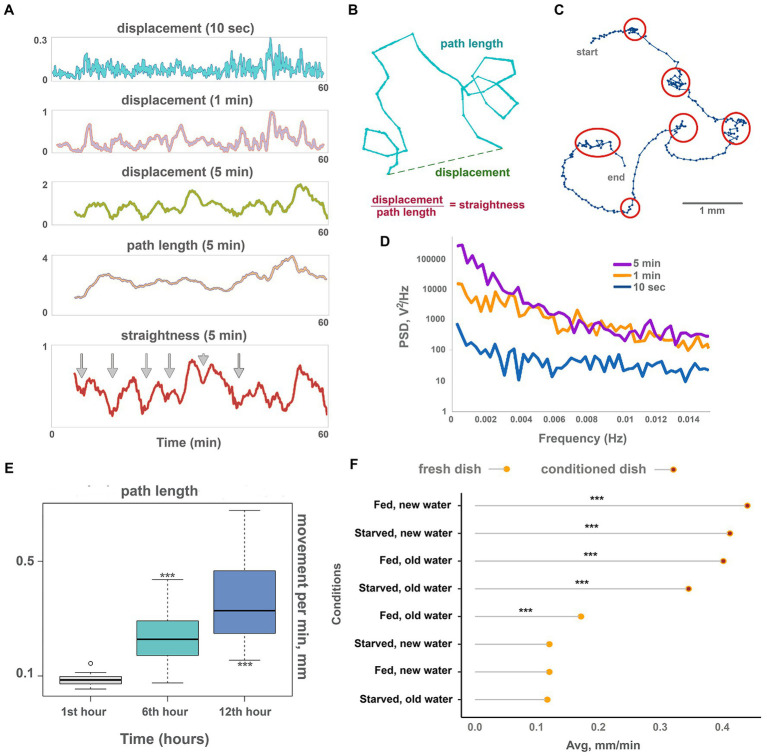
Oscillations and long-term dynamics during normal locomotion. **(A)** The plot of displacements (how far out of starting place an animal is located) of a single animal for 10 s, 1 min, and 5 min periods, plus track length and track straightness (displacement/length ratio) for 5 min periods observed for 60 min on conditioned Petri dish. While 10-s displacements are chaotic, oscillations emerge in 1 min and are clearly observed in 5 min displacements. Larger scale oscillations of 5 min displacement with a period of about 6–8 min coincide with fluctuations of path straightness. The path length is more stable over time and does not show regular oscillations. Arrows denote chaotic “marking time” periods every 6–8 min when track straightness is reduced. **(B)** Scheme showing displacement (how far out of an animal’s starting position), path length, and straightness. **(C)** Track of the *Trichoplax* with dots marking an animal’s positions every 10 s, recorded for 40 min—it is the same specimen as in **A**. Six periods of decreased locomotory speed (dense dots) are highlighted with red circles and correspond to straightness minima marked by arrows in **A**. Note that periods of decreased speed coincide with sharp turns of the track. See also Supplementary Videos S3. Animal size is about 0.5 mm. **(D)** Plot of power spectral density (PSD) of 10 s, 1 min, and 5 min displacements on conditioned Petri dish (average of 10 animals). PSD of 10-s displacements is nearly uniform (characteristic of white noise), while PSD of 1 min and especially 5 min displacements show pronounced low-frequency fluctuations (below 0.004 Hz, or periods above 250 s). **(E)** The speed of locomotion of the *Trichoplax* is gradually accelerated over 12 h. Animal locomotion and tracks were recorded at 60-min intervals. Asterisks denote significant differences in the locomotion speed from initial observations during the 1st hour (Welch *t*-test, ^***^p < 0.001) N animals = 20. **(F)** Factors affecting the long-term acceleration of *Trichoplax adhaerens* on glass dish: animal starvation, water, and glass freshness. Data are presented as median ± SD. *N* = 33, 36, 37, 34, 32, 35, 33, 35. Locomotion is slow on a fresh glass dish and accelerated on the conditioned (“old”) glass where placozoans (same or another specimen) crawled for 24 h before. The speed of locomotion was recorded for 120 min. Asterisks denote a significant difference in speed from the control condition (new dish, new water, well-fed animals; Welch *t*-test, ^***^p < 0.001).

All tested animals showed spontaneous long-term oscillations of locomotory patterns under control conditions, even without algae or any other substrate. This is surprising because previous studies described *Trichoplax* locomotion as Brownian, which does not imply regular endogenous oscillations (see Discussion). We quantified this long-term rhythmicity, with a period of about 8 min, by power spectral density analysis using displacement parameters (Figures 2A,D). With 10-s interval displacement, the spectrum was nearly uniform (similar to white noise), while 1-min and especially 5-min displacement intervals showed an excess of low frequencies below 0.004 Hz (periods above 250 s).

Displacement can be expressed as the product of two values: path length and straightness (Figure 2B). Figure 2A shows that long-term oscillations in displacement were mirrored by the path straightness, whereas path length values were more constant (Figure 2A). Thus, fluctuations in displacement are associated with changes in turn patterns but not speed. In other words, *Trichoplax* locomotion is reminiscent of the “Viennese waltz,” where “dancers” switch between straight motion and turn in one place (Supplementary Video S3).

Long-term recordings (24 h) also showed that locomotion speed increased over time. Locomotion was relatively slow during the first 2 h of their placement into experimental dishes (below 0.2 mm/min), gradually accelerating in the first 12 h. After that, the animal speed reached a plateau at about 0.4 mm/min (Figure 2E; Supplementary Video S4). The trajectory shapes did not change. We have hypothesized that the observed gradual increase in locomotion could be either mediated by animals’ internal state (e.g., starvation level) or by changing environment (chemical composition of water and glass surface).

To choose between these possibilities, we have measured the locomotion of animals that were first starved for 24 h and then transferred to a new clean dish vs. animals moved a to a conditioned dish where other *Trichoplax* specimens were crawling for 24 h before tests. We also tried combinations of fresh seawater on the conditioned dish, and conditioned seawater in a clean dish. To achieve this, we transferred seawater from the conditioned dish to the clean dish or gently poured fresh artificial seawater (ASW) into the emptied conditioned dish before placing the animals.

Combined, these experiments have shown that *Trichoplax* locomotion is significantly faster (about three times) on the conditioned surfaces compared to clean dishes, regardless of water freshness and animal starvation state (Figure 2F). These results suggest that the observed acceleration of locomotion over time is mediated by changing substrate by animals. For example, it is well-known that *Trichoplax* can release mucus during locomotion (Mayorova et al., 2019) and accumulation of the mucus can increase the speed on the conditioned dishes.

#### Ca-dependent secretion of signal molecules can contribute to observed behavioral dynamics in placozoans

Low-frequency oscillations of feeding and locomotory patterns might be a result of complex intercellular chemical interactions (e.g., volume-type transmission) among different cell types in placozoans. In experiments on neural systems, polysynaptic inputs can be eliminated (or significantly suppressed) by high [Mg^2+^] concentrations, where these ions, attracted to negatively charged phosphate residues of membrane phospholipids, caused functional membrane hyperpolarization and increased thresholds for generation of action potentials, depolarization-induced secretion and eventually chemical transmission in central and peripheral synapses (Del Castillo and Engbaek, 1954; Hutter and Kostial, 1954; Arossa et al., 2022). This phenomenon and seawater with high MgCl_2_ concentration are widely used for anesthesia and relaxation of marine invertebrates (Arossa et al., 2022). Decreased concentrations of [Ca^2+^] also suppress calcium-dependent exocytosis, including exocytosis of transmitters in all types of neurons and other secretory cells. Thus, if the integration of locomotory functions in *Trichoplax* is dependent on action potentials or calcium-dependent exocytosis, we can expect its disruption at high [Mg^2+^] or low [Ca^2+^] concentrations.

**Figure 3:**
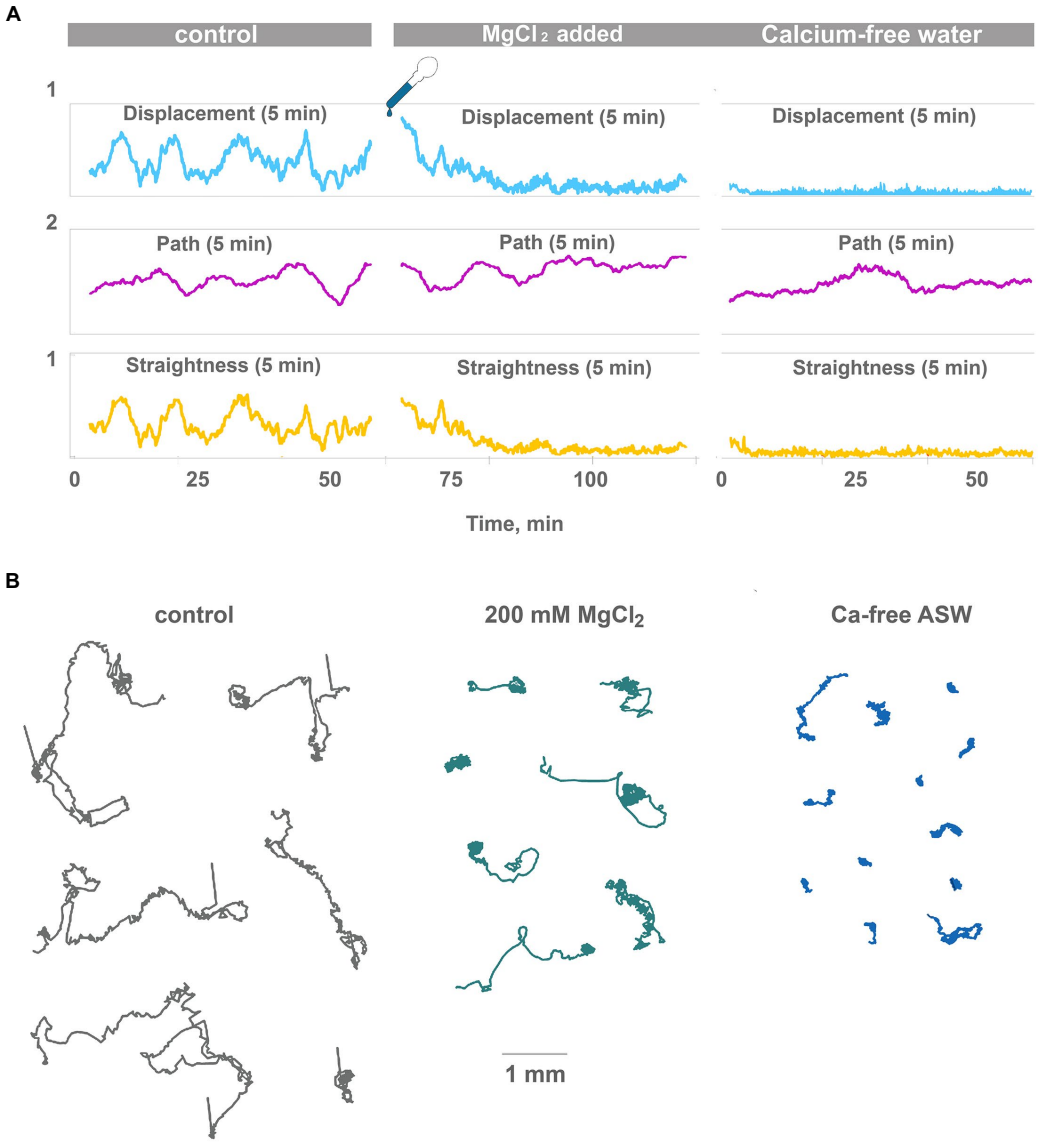
High Mg^2+^ or low Ca^2+^ disrupts coherent locomotion and oscillations. **(A)** Representative plot of displacement, track length, and track straightness (displacement/length ratio) for 5 min periods for a single animal observed for 120 min on a clean Petri dish in normal seawater and after the addition of 200 mM MgCl_2_ (corrected for osmolarity). Oscillations of straightness and displacement present in control conditions (compare Figure 2A) and gradually disappear after 20 min in 200 mM MgCl_2_. However, the 5-min path is unchanged. Therefore, animals continue ciliated locomotion but lose the ability to maintain one direction. Representative plot of displacement, track length, and track straightness for 5 min periods for single animal observed for 60 min in low-Ca seawater. Displacement and straightness are close to zero immediately after a drop in Ca^2+^ concentration, but track length is similar to control. Coherent directed movement is quickly lost, compared to 20 min transition period in 200 mM MgCl_2_. **(B)** Tracks of *Trichoplax* in normal seawater (45 mM Mg^2+^, 10 mM Ca^2+^ as control), after adding 200 mM MgCl_2_, and in low-Ca seawater (1 mM Ca^2+^), respectively. Tracks were recorded for 60 min.

After adding ASW with 200 mM [Mg^2+^] directional locomotion of *Trichoplax* gradually disappears. After 20 min in high [Mg^2+^], animals only rotate and wobble in one place (Figures 3A,B; Supplementary Video S5). Low-frequency oscillations in displacement and straightness were decreased 5–10 times compared to the control, but the 5-min path is unchanged. In other words, cilia beating continued unchanged on the level of individual cells, but coordination between cells, required for coherent locomotory patterns, was wholly disrupted (Figure 3B). The decrease of [Ca^2+^] concentration from normal 10 mM in the seawater to 1.5 mM had similar but faster effects (Figures 3A,B; Supplementary Video S6). Directional locomotion also stopped after 1 min in 1.5 mM [Ca^2+^]; *Trichoplax* continued to turn and wobble in one place like in 200 mM [Mg^2+^] (Figure 3B; Supplementary Video S6).

In summary, these experiments confirm the hypothesis that Ca-dependent secretion of signal molecules can contribute to observed low-frequency oscillations of behavior in placozoans.

### Pharmacology of amino acids’ signaling

#### L-/D-glutamate and L-/D-aspartate initiate feeding-like cycles even on a clean substrate

**Figure 4:**
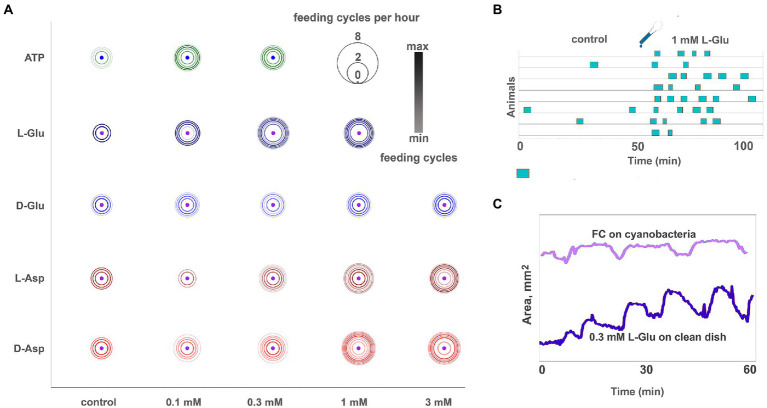
Glutamate, aspartate, and ATP induce reactions similar to feeding cycles on algal biofilm. **(A)** Bubble plot comparison of various ATP, L- and D-glutamate concentrations and L- and D-aspartate in inducing feeding-like cycles. Pharmacological activity decreases in the following order: L-Glu > D-Asp > L-Asp > D-Glu. Unlike other tested amino acids, the D-Glu response is not dose-dependent (the difference between adjacent concentrations is insignificant). Feeding cycles for 3 mM L-Glu are not shown because this concentration elicits prolonged (30–90 min) arrest of locomotion and nearly permanent body expansion instead of regular 5–10 min feeding cycles (see Supplementary Video S8). Scale: feeding cycles per hour, the color bar was normalized: black—max counts, light grey—min counts. **(B)** Diagram of feeding-like activity of *Trichoplax adhaerens* on the clean glass dish. In control, only a few occasional cycles are observed. After adding 1 mM L-glutamate, *Trichoplax* exhibit repeated feeding cycles similar but not identical to those observed on natural food substrates. **(C)** The plot of body area for representative animals feeding on cyanobacteria (light blue line) and clean glass upon addition of 0.3 mM L-Glu (dark blue line). Feeding-like cycles induced by L-Glu are shorter than natural feeding cycles (7–10 min vs. 10–20 min), lack a ‘digestion phase’ with churning movements (plateau on the area plot), and have a more extended body contraction phase. In glutamate-induced feeding-like cycles, body contraction is often incomplete, and body area increases over consecutive cycles.

Enantiomers of glutamate (Glu) and aspartate (Asp) are among the most abundant metabolites in basal metazoans. However, their functional roles in these animals are elusive at this moment (Moroz et al., 2020b). In placozoans tested on a clean glass dish, both enantiomers of glutamate and aspartate induced reactions resembling feeding cycles on algal biofilms (Figures 4B,C; Supplementary Video S7). However, Glu- and Asp-induced cycles are shorter than natural feeding cycles (8–12 and 10–20 min/cycle, respectively), lack a churning phase, and their contraction phase is prolonged and incomplete. In a series of 3–4 Glu- or Asp-induced feeding-like cycles, the animal surface area increases from the first cycle to the next (Figure 4C).

The same type reduced feeding-like cycles without the churning phase are observed occasionally in clean glass dishes without any substances. 1–2 h after the addition of Glu or Asp., these cycles disappear, and animals continue normal locomotion.

Enantiomers of Glu and Asp had different behavioral effectiveness, increasing in order D-Glu → L-Asp → D-Asp → L-Glu. D-Glu is the least active of all; it induces 1.5–2 feeding-like cycles/h on average (Figure 4A). The effect of D-Glu is not dose-dependent in the tested range of concentrations from 0.1 to 3 mM (Figure 4A), and other isomers have dose-dependent effects (Figure 4A). The maximum possible effect, namely, animals continuously exhibiting feeding-like cycles (~ 4.5/h), is observed for ≥ 0.3 mM L-Glu, ≥ 1 mM D-Asp., and 3 mM L-Asp (Figure 4A).

A distinct effect was observed in the presence of 3 mM L-glutamate. *Trichoplax* expands and retains the same expanded shape for 2–3 h. During this time, animals exhibit elements of feeding-like cycles (stops and resuming locomotion, attachment, and detachment of edges) but do not perform contractions (Supplementary Video S8). After 2–3 h, animals folded into lumps, stopped moving, and then dissociated into separate cells, and die. 3 mM of L- and D-Asp or D-Glu did not show such adverse effects even after 24 h of incubation. Of note, no L-/D-glutamate and L-/D-aspartate initiate effects were observed when animals were already feeding on algae (data not shown).

#### ATP positively and negatively modulates feeding at different time scales

ATP and L-glutamate are the two major metabolites in the cytoplasm of most cells (Moroz et al., 2021a). They are released in media upon cell damage, could be sensed by other cells, and used to trigger defensive/regenerative or feeding responses. This injury-related sensing of L-glutamate and ATP could be exaptations for early neural signaling (Moroz et al., 2021b). Therefore, we tested *Trichoplax’s* behavioral responses to ATP.

**Figure 5:**
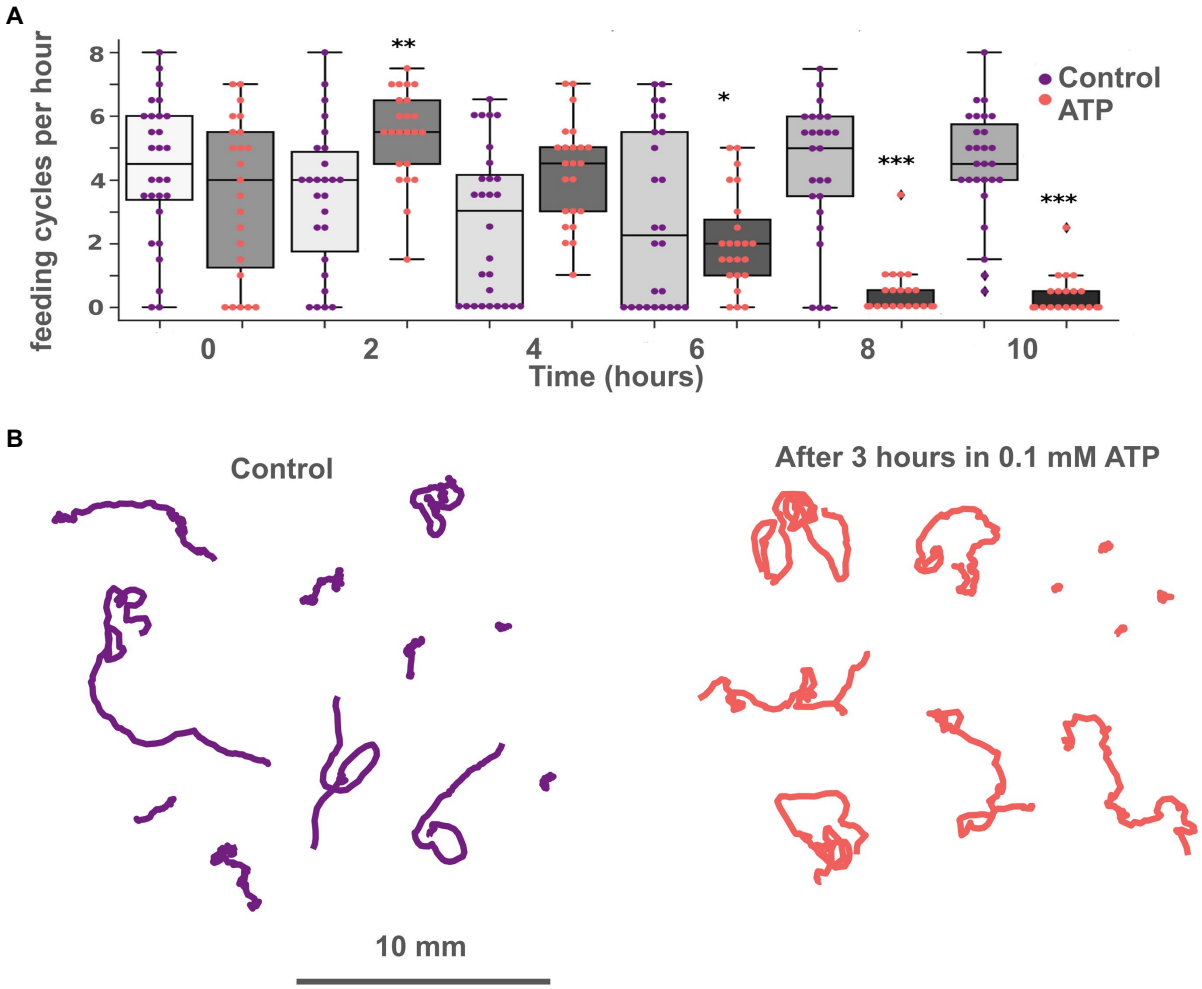
ATP exhibits long-term suppression of feeding and does not affect locomotion. **(A)** Bar chart of feeding activity of *Trichoplax* on green algae biofilms in normal conditions and with 0.1 mM ATP. Adding ATP to the dish with green algae increases ‘feeding’ activity at first and decreases after 6 h. In the experimental dish, ATP was added at 2 h. Asterisks denote a significant difference in feeding activity from the control (Welch *t*-test, ^*^p < 0.05, ^**^p < 0.01, ^***^p < 0.001). N animals = 37 and 28 for ATP and control, respectively. **(B)** Tracks of animals incubated in 0.1 mM ATP for 3 h (right) and in control (left). No significant differences in track length, straightness, or displacement were found.

On the clean glass, the effect of ATP was very similar to glutamate but weaker. 3–5 min upon the addition of ATP, a series of 2–3 feeding-like cycles were observed (Figure 4A). These feeding-like cycles were shorter than naturally occurring feeding cycles on algal biofilms and lack the “churning” phase, the same as observed after L-glutamate addition. In contrast to Glu, these cycles ceased after about 1 h of incubation in ATP. After that, animals behaved similarly to controls (Figure 5B). Concentrations of 0.1 and 0.3 mM of ATP had the same magnitude of effects.

On the algal biofilm, the effects of ATP were more complex and prolonged. At first 1–2 h, feeding of *Trichoplax* was activated compared to control (unless they were not eating all the time before). But after 8–10 h of incubation with ATP, feeding was ceased almost entirely, while control animals continued to eat (Figure 5A). Similar long-term feeding suppression was observed for GABA (see below) but not for L-/D-glutamate, D-/L-aspartate.

#### Glycine and GABA suppress feeding cycles.

**Figure 6:**
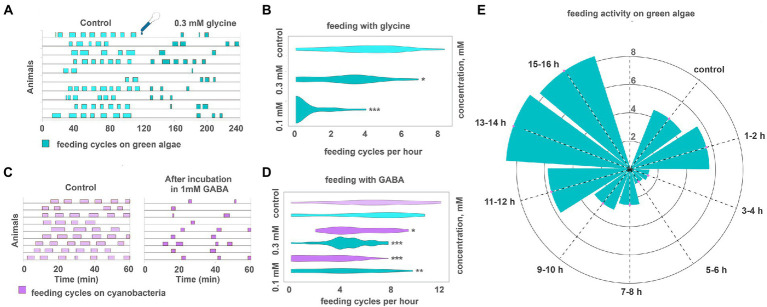
GABA and glycine modulate feeding cycles in *Trichoplax adhaerens*. **(A)** After adding 0.3 mM glycine, the number, and frequency of feeding cycles on green algae biofilm reduced approximately threefold compared to the control. **(B)** The effect of glycine on feeding activity is dose-dependent. 0.1 mM reduces the number of feeding cycles by 26%, 0.3 mM—by 86%. Asterisks denote a significant difference in feeding activity from the control (Welch *t*-test, ^*^p < 0.05, ^***^p < 0.001). N animals = 65, 36, 29. **(C)** After 20 h in 1 mM GABA **(C**, right**)**, the number and frequency of feeding cycles reduced approximately threefold compared to the control [after 20 h in pure artificial seawater (ASW)] **(C**, left**)**. In control, *Trichoplax* exhibit feeding cycles every 10–15 min. **(D)** GABA decreased feeding activity on green algae and cyanobacterial biofilms in concentrations of 0.3 and 1 mM. Asterisks denote a significant difference in feeding activity from the control (Welch *t*-test, ^*^p < 0.05, ^**^p < 0.01, ^***^p < 0.001). N animals = 80, 64, 27, 26, 47, 40. **(E)** After adding 0.3 mM GABA, feeding activity decreased on the green algal mat (*T. marina*) after 1–2 h from the starting point and recovered after 11–12 h of incubation. N animals = 10.

Glycine suppressed placozoan feeding on algal biofilms. This effect was relatively fast, on the order of 10–20 min (Figure 6A; Supplementary Video S9). Effect was dose-dependent: 0.1 mM glycine reduced the frequency of feeding cycles by 26%, 0.3 mM—by 86% (Figure 6B). Higher concentrations were not tested because toxicity was observed upon prolonged (24 h) exposure to 0.3 mM glycine. However, the effect of 1–2-h exposure to 0.3 mM glycine was reversible as animals resumed feeding at an average rate 15–30 min after glycine washout (data not shown).

Feeding was suppressed after prolonged incubation in GABA. Animals preincubated in 1 mM GABA for 20 h before relocation to algae showed 60% fewer feeding cycles per hour than average (Figures 6C,D). The dynamics were different if animals were incubated in GABA in a dish with algae. Feeding was normal in the first 2 h with GABA, suppressed between 3 and 6 h after GABA addition, and then recovering phase, reaching full recovery 14–16 h from GABA addition (Figure 6E; Supplementary Video S10). This difference in dynamics might result from form algae-meditated degradation of GABA.

#### Effects of amino acids on placozoan locomotion

**Figure 7:**
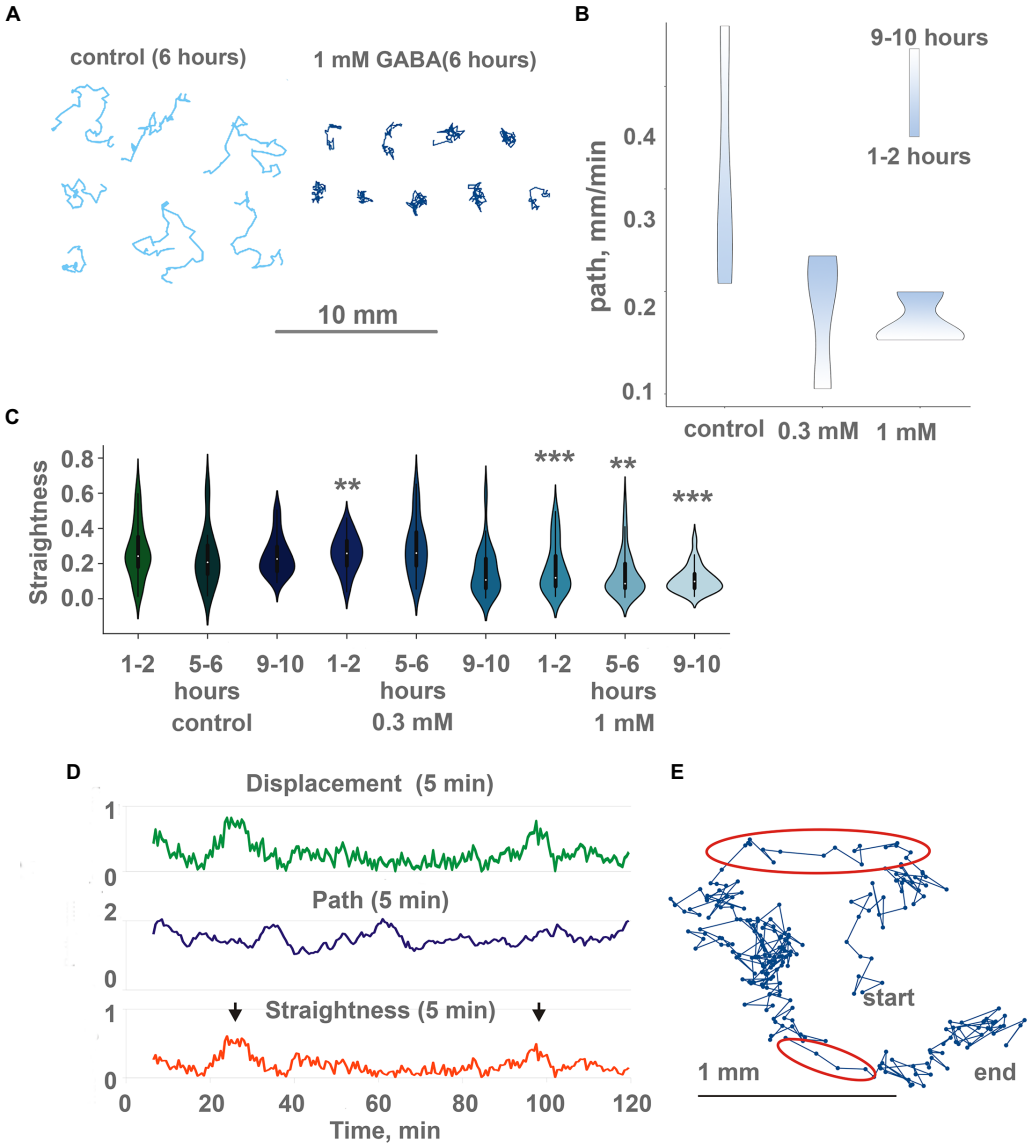
GABA modulate locomotion in *Trichoplax adhaerens*. **(A)** Tracks of animals incubated for 6 h in 1 mM GABA (right) and ASW (left). After GABA incubation, tracks are shorter and more squiggly compared to the control. Tracks were recorded for 60 min. **(B)** in native conditions, animals show increasing path length through time (from starting point to the destination), the additional concentration of GABA (0.3 and 1 mM) changes, and decreasing path. Individuals placed in the same locations for a long time (9–10 h). Scale bar: blue—1-2 h, white—9–10 h. **(C)** During incubation with GABA, track straightness gradually decreases, while in control, it is unchanged. Asterisks denote a significant difference in track straightness from the control (Welch *t*-test, ^**^p < 0.01, ^***^p < 0.001). *N* = 98, 50, 74, 29, 46, 69, 35, 44, 72. **(D)** Representative plot of displacement (green), track length (blue), and track straightness (red, displacement/length ratio) for 5 min periods for a single animal observed for 120 min on a conditioned Petri dish after 10 h in 1 mM GABA. Straightness and displacement are mostly low except for two periods at 25 and 100 min (arrows). **(E)** Track of the same *Trichoplax* as in Figure 6D with dots marking positions every 10 s, recorded for 120 min. Two periods of nearly straight movement are highlighted with red ellipses and correspond to straightness maxima marked by arrows in Figure 6D. Compare with a track of control animal in Figure 2C.

GABA (0.3-1 mM) had a profound inhibitory effect on *Trichoplax* locomotion after long (6–24 h) incubation. Animal tracks became extremely convoluted in the presence of GABA compared to the control (Figure 7A; Supplementary Video S11). Furthermore, slow oscillations of locomotory parameters (5 min displacement and straightness) with a period of about 6–10 min, observed in control testes, disappeared in the presence of GABA. Occasional 2–5 min long bursts of directed gliding were observed, interleaved with long turns and wobbling in one place (Figures 7D,E; 2 episodes of directed gliding in 120 min). Average track straightnesses were decreased from 0.27 to 0.12 after 12 h in 1 mM GABA on the clean dish (Figure 7C), and to a lesser extent on the conditioned dish (Supplementary Figure S1B). Suppression of directed gliding was partially similar to 200 mM Mg^2+^.

The second effect of GABA on locomotion was a decreased average speed of animals on conditioned glass. If animals were placed on a conditioned dish with GABA, their average speed decreased twofold over 12 h (Figure 7B). On a clean dish with GABA, speed was uniformly low during the 24 h of recording (Supplementary Figure S1A), while in control, they accelerated from ~ 0.15 to ~ 0.5 mm/min in 12–20 h (Figure 1F).

**Figure 8:**
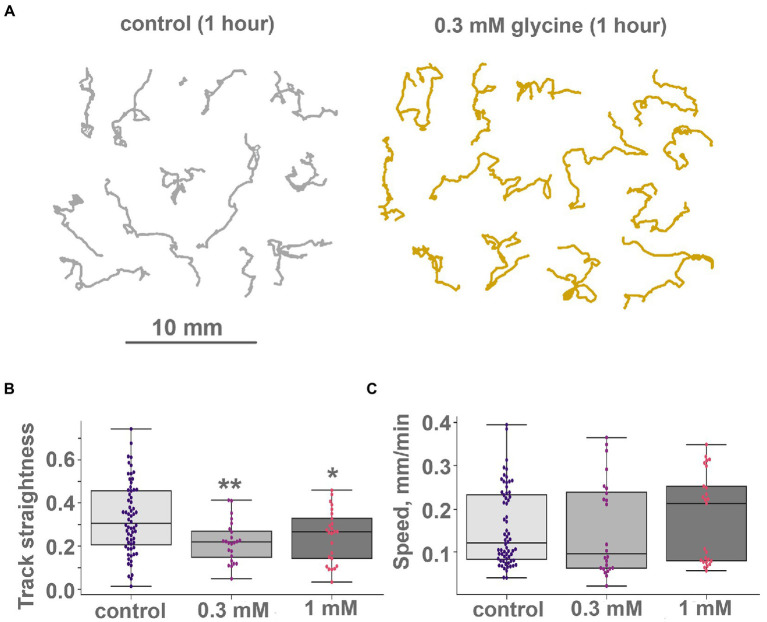
Glycine modulate locomotion in *Trichoplax adhaerens*. **(A)** Tracks of animals incubated for 1 h in 0.3 mM glycine (right) and ASW (left). After adding glycine, incubation tracks are squigglier than the control, but their length is unchanged. Tracks were recorded for 60 min. **(B)** and **(C)** Glycine modulates turn patterns but does not affect locomotory speed. Diagrams of track straightness **(B)**, path length divided by distance between start and end) and crawling speed **(C)**, (mm/min) for the control, 0.1 and 0.3 mM glycine. Glycine decreases track straightness but does not affect locomotion speed. Asterisks denote a significant difference in track straightness from the control (Welch *t*-test, ^*^p < 0.05, ^**^p < 0.01). All three speed values are not different (Welch *t*-test, value of *p* > 0.05). N animals = 64, 24, and 25, respectively.

Locomotion on clean glass was also subtly affected by glycine. The speed of animals’ locomotion was unchanged (Figure 8C), but track shapes became more convoluted (Figure 8A; Supplementary Video S12). Quantitatively, the 5-min straightness index and 5-min path and displacement were not changed upon glycine addition; their long-term oscillations were not changed either. However, 60-min straightness was decreased significantly upon glycine addition. On the clean glass, 60 min straightness index (ST, see “Materials and methods”) was 0.32 ± 0.16 (median ± SD) in control. After adding 0.1 mM glycine, 60-min ST decreased to 0.22 ± 0.09 (Figure 8B). This difference was statistically significant at *p* < 0.01. 0.3 mM of glycine caused a slightly weaker effect (ST = 0.24 ± 0.12, significantly different from the control at *p* < 0.05).

### Molecular bases of amino acids’ sensing in placozoans

#### Receptors

**Figure 9:**
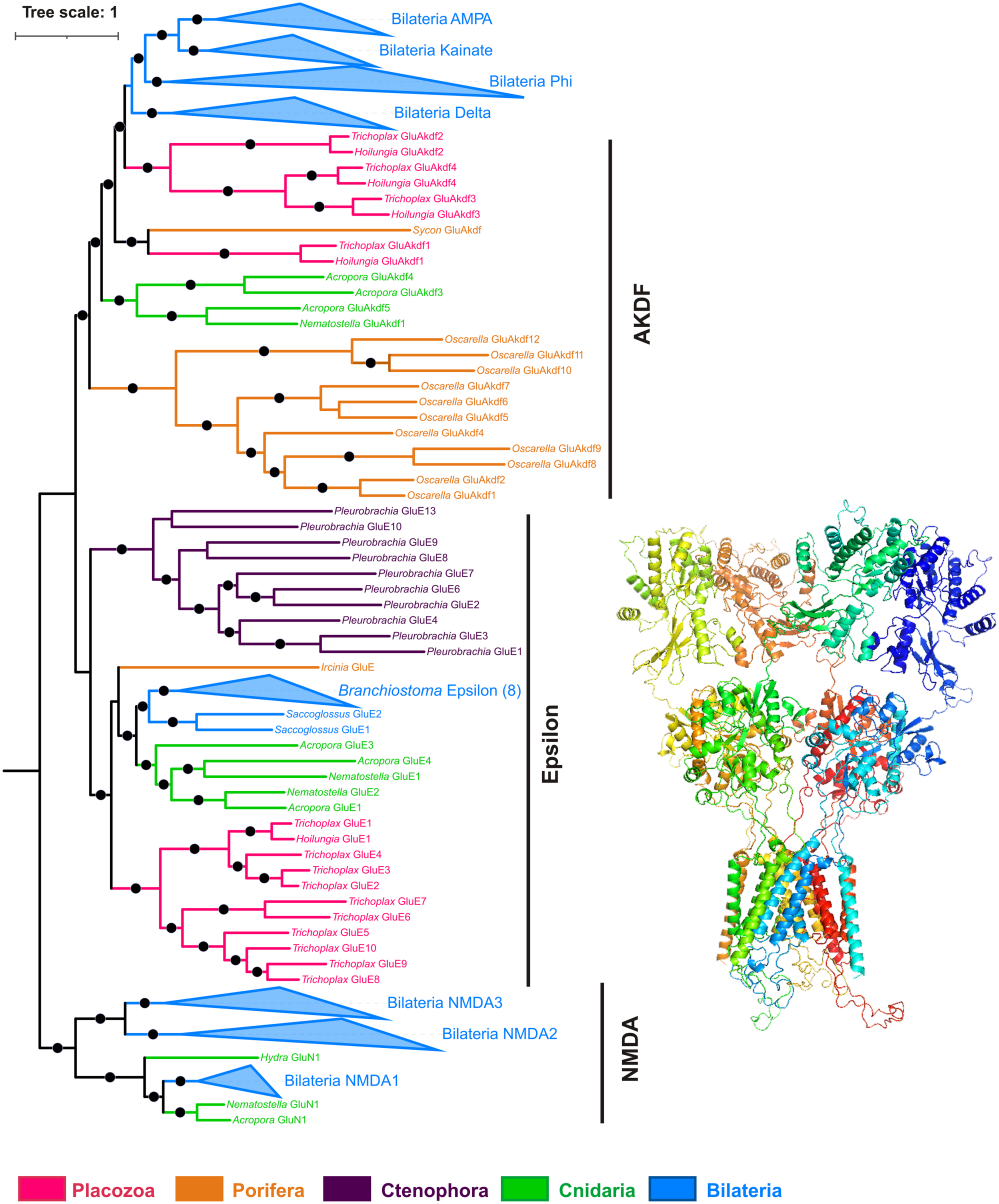
Phylogeny of ionotropic glutamate receptors (iGluRs). Bayesian phylogenetic tree of iGluRs has well-supported clades corresponding to most metazoan iGluR families (NMDA, AMPA, kainate, Delta, Phi, Epsilon), plus a paraphyletic group of AKDF-type receptors. *Trichoplax* has 14 iGluRs (4 AKDF and 10 Epsilon), and *Hoilungia* – has 5 iGluRs (4 AKDF and 1 Epsilon). Placozoan AKDF receptors split into two independent clades. Black dots mark highly supported nodes (Bayesian posterior probability > 0.75). Full uncollapsed trees with numeric support values can be found in Supplementary Figure S3.

**Figure 10:**
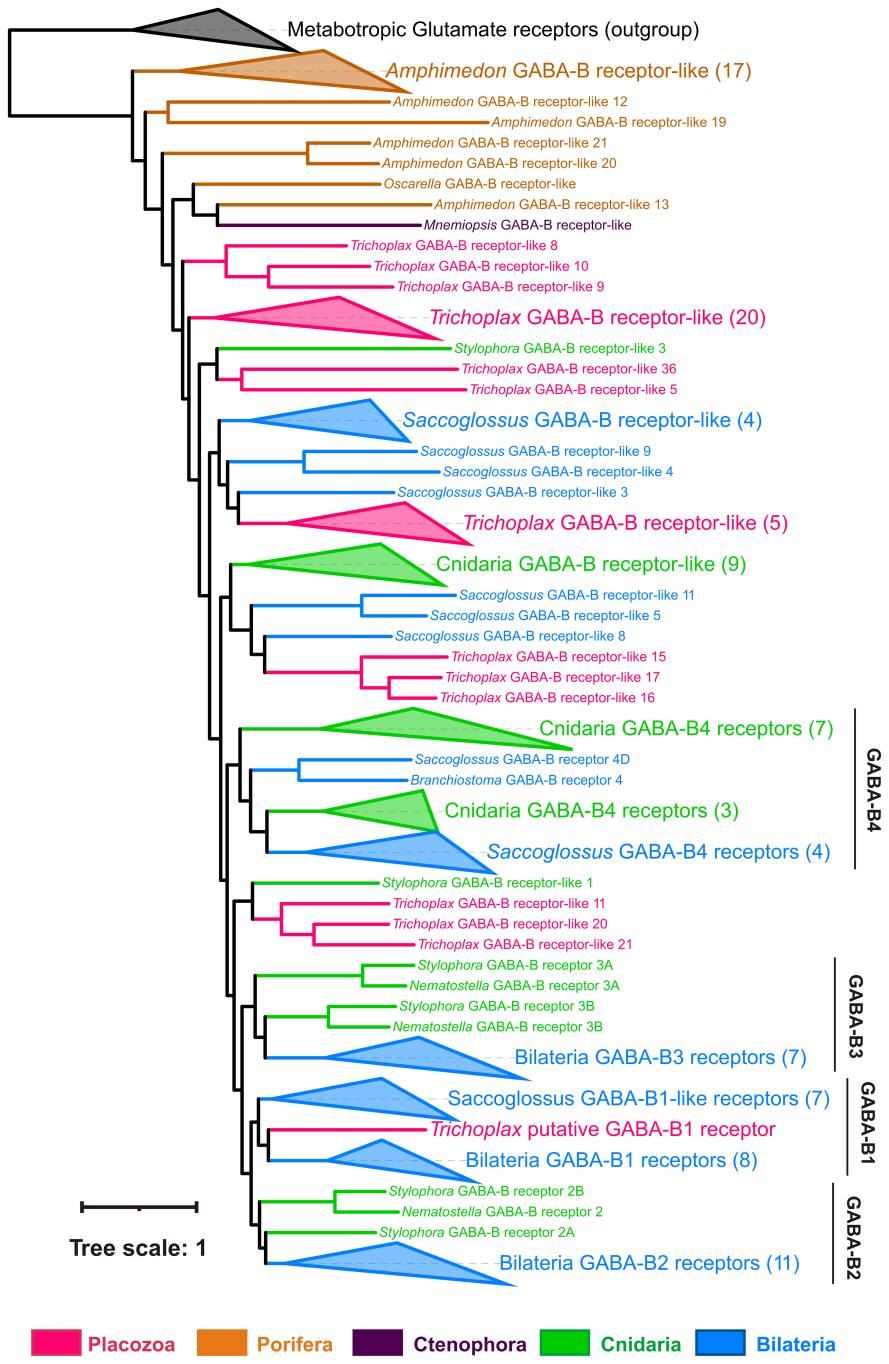
Phylogeny of GABA-B-like receptors: *Trichoplax* has 37 GABA-B-like receptors, more than any other analyzed species (Maximum likelihood phylogenetic tree). One placozoan receptor is orthologous to vertebrate GABA-BR1 and is the first candidate for sensing GABA. Placozoan GABA-B-like receptors form seven separate clades in the tree. Similarly, cnidarians and hemichordate *Saccoglossus* also have numerous GABA-B-like receptors (16 in *Stylophora*, 29 in *Saccoglossus*), comprising several different clades. This tree structure suggests high diversity of GABA-B-like receptors in basal metazoans and their subsequent loss in many bilaterian lineages. Black dots mark highly supported nodes (bootstrap support > 0.75). Full uncollapsed trees with numeric support values can be found in Supplementary Figure S4.

**Figure 11:**
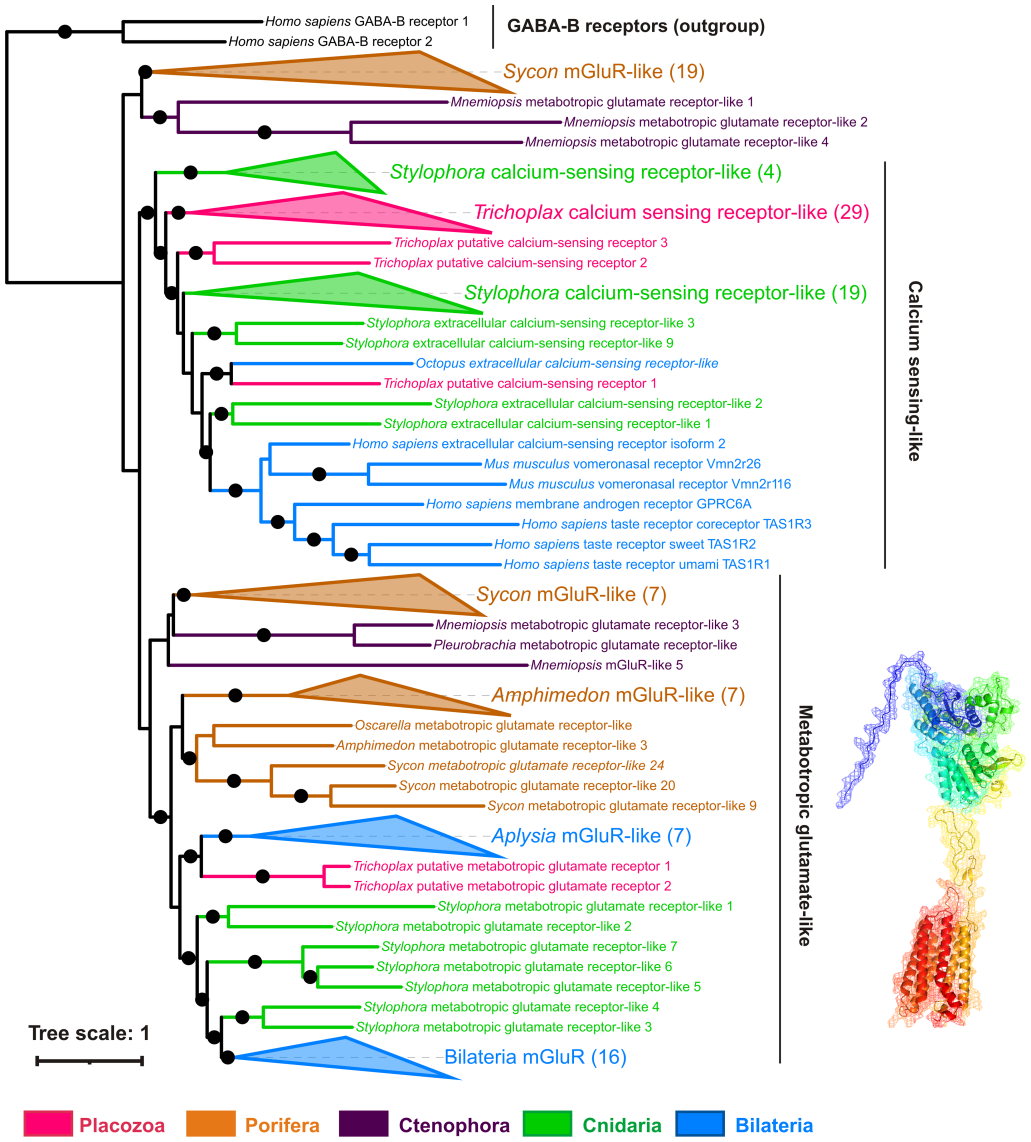
Phylogeny of metabotropic glutamate receptors (mGluRs): *Trichoplax* has 34 receptors of this highly diverse superfamily. Maximum likelihood tree shows mGluRs and related receptors such as extracellular calcium-sensing (CaSR), taste, and vomeronasal receptors. *Trichoplax* mGluR-related receptors form 4 separate clades in the tree. Two *Trichoplax* receptors are sisters to a clade of *Aplysia* mGluR-like receptors; the other three clades are more closely related to calcium-sensing, taste, and vomeronasal receptors. Similarly, cnidarians and calcisponge *Sycon* have numerous mGluR-like receptors (30 in *Stylophora*, 29 in *Sycon*), forming several clades. This tree structure suggests that the divergence of mGluRs and CaSR-like receptors predates the radiation of basal metazoan phyla. Black dots mark highly supported nodes (bootstrap support > 0.75). Full uncollapsed trees with numeric support values can be found in Supplementary Figure S5.

How and why observed effects of amino acids and ATP are mediated? We have analyzed the presence of receptors and relevant vesicular transporters involved in glutamatergic, GABAergic, and purinergic signaling within the *Trichoplax* genome (Srivastava et al., 2008). We have found 85 amino acid receptor genes, which encode14 ionotropic glutamate (iGluRs), 34 metabotropic glutamate-like (mGLuRs), and 37 metabotropic GABA-like (GABA-BRs) receptors (Supplementary Table S1). This is the richest repertoire of amino acid receptors among all analyzed animals, from sponges to humans. Humans have 25 amino receptors in these families (15 iGluR, 8 mGluR, and 2 GABA_BR), sponges *Amphimedon* and *Sycon*—0, 8, 21, and 1, 28, 0, respectively. This is due to remarkable lineage-specific expansions of mGluRs, GABA-BRs, and Epsilon-type iGluRs in Placozoa. Ionotropic GABA-A and glycine receptor genes were not found in *Trichoplax* genome. Similar independent radiation events were found in the coral *Stylophora* and the hemichordate *Saccoglossus* (Figures 9–11; Supplementary Table S1). Ctenophores have an expansion of Epsilon iGluRs (lost in many bilaterian lineages) but very few metabotropic receptors. Homosclerid sponge *Oscarella* has an expansion of AKDF-type iGluRs but only single mGluR and GABA-BR. In contrast, in all analyzed species of demosponges and calcisponges (*Amphimedon*, *Ephydatia*, *Ircinia*, and *Sycon*), we noted a dramatic expansion of metabotropic receptors with a few cases of iGluRs (Figures 9–11; Supplementary Table S1).

Most analyzed *Trichoplax* receptors are not closely related to well-annotated mammalian receptor families. But *Trichoplax* does have one ortholog of GABA-BR1, two metabotropic glutamate-type receptors, and one encoding extracellular calcium-sensing receptors (Figures 10, 11; Supplementary Table S1).

All placozoan iGluRs belong either to Epsilon (10 members) or AKDF (4 members) families. On the phylogenetic tree (Figure 9; Supplementary Figure S3), one can see that placozoan AKDF receptors diverged in parallel with sponge and cnidarian clades and clustered with receptors from the calcareous sponge *Sycon*. This tree topology suggests the ancient radiation of AKDF receptors before major animal phyla diversification and reflects retaining this ancestral diversity in Placozoa.

Placozoan GABA-B receptors form seven independent clades across the phylogenetic tree and interleaved with multiple clades of cnidarian and hemichordate GABA-BRs (Figure 10; Supplementary Figure S4). This implies the rich ancestral diversity of GABA-B-like receptors that was lost in most Bilateria but preserved in hemichordates and basal metazoans.

Metabotropic glutamate-like receptors in Placozoa belong to four distinct clades (Figure 11). One is orthologous to molluscan mGluRs; three others are more related to extracellular calcium, taste, and vomeronasal receptors (Supplementary Figure S5).

Textbook glycine receptors are chloride-gated (inhibitory) members of the ionotropic pentameric Cys-loop receptor family. Cys-loop receptors are absent in the sequenced *Trichoplax* genome. However, glycine can act as a co-agonist of NMDA-type iGluRs (Ramos-Vicente et al., 2018) and was shown as the main ligand of two Epsilon-type excitatory iGluRs in Ctenophora and *Branchiostoma* (Alberstein et al., 2015; Ramos-Vicente et al., 2018). Therefore, some placozoan iGluRs can function as excitatory ionotropic glycine receptors.

**Figure 12:**
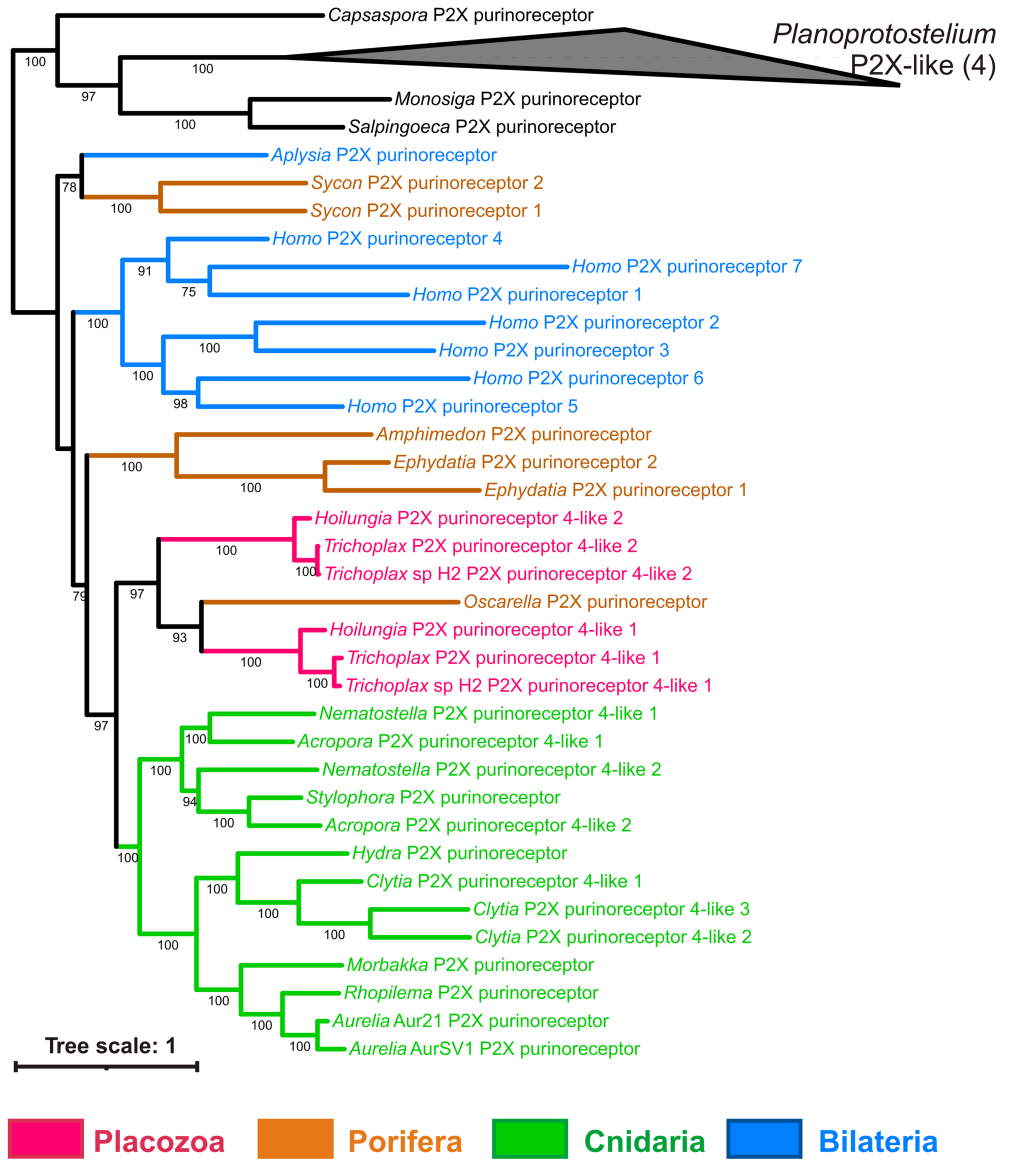
Bayesian phylogenetic tree of ionotropic purine (P2X) receptors. *Trichoplax* has 2 P2X receptors, and representatives of other invertebrate lineages have from 1 to 3 P2X receptors encoded in their genomes. Bayesian posterior probabilities of nodes (> 0.5) are shown.

ATP receptors are classified into ionotropic P2X and metabotropic P2Y (Abbracchio and Burnstock, 1994). P2X receptors were found in the *Trichoplax* genome (Srivastava et al., 2008) and localized immunohistochemically to fiber cells of the middle cell layer (Smith et al., 2014). Neither we nor Srivastava et al. (2008) were able to find any P2Y receptors in *Trichoplax*. Our phylogenetic analysis of P2X receptors (Figure 10) shows that all placozoans have two P2X genes most similar to their respective homologs from the sponge *Oscarella*. Cnidarians and sponges also have from 1 to 3 P2X receptors (Figure 12; Supplementary Table S1).

#### Transporters

Transmitters, such as amino acids and ATP, can be accumulated in secretory vesicles by specialized subfamilies of vesicular transporters. Vesicular transporters for both glutamate and ATP belong to the SLC17 family of membrane transporters together with sialic acid transporter (SLC17A5, also known as sialin), phosphate and urate transporters (SLC17A1-A4).

**Figure 13:**
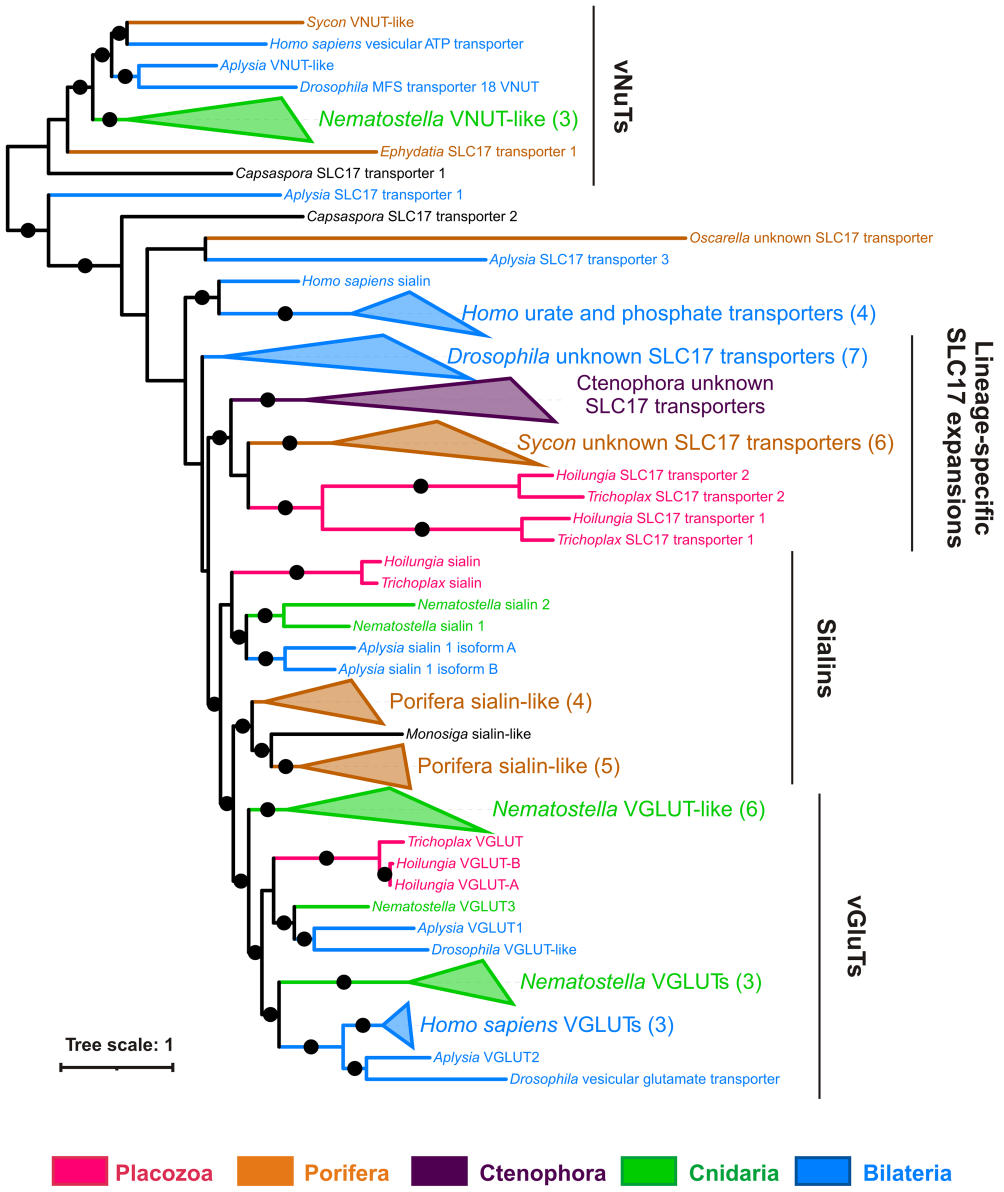
*Trichoplax* has a gene encoded putative vesicular glutamate transporter but no vesicular ATP transporters. Bayesian tree of vesicular glutamate and related transporters (SLC17 family) is shown. The superfamily of SLC17 transporters includes transmembrane carriers for various anions (phosphate, nucleotides, glutamate, and sialic acid). There are three well-defined clades of vesicular glutamate (vGluTs), sialins, and vesicular nucleotide (vNuT) transporters, plus several independent branches with lineage-specific expansions. Placozoan (*Trichoplax* and *Hoilungia*) transporters fall within vGluT and sialin clades, plus one separate clade of unknown substrate specificity. Full uncollapsed trees with numeric support values can be found in Supplementary Figure S6.

Figure 13 shows the phylogenetic tree of the SLC17 family with well-supported clades of vesicular glutamate transporters (vGluTs), sialins, and vesicular ATP transporters (vNuTs), as well as examples of lineage-specific radiation events. Placozoan SLC17 transporters form three clades belonging to vGluT, sialins, and the uncharacterized subfamily of ctenophore and sponge transporters. Therefore, *Trichoplax* does have a vesicular glutamate transporter and might utilize components of canonical glutamatergic transmission. VNuT clade contains predicted proteins from Porifera, Cnidaria, Bilateria, and the unicellular eukaryote *Capsaspora* (Figure 13). However, vNuT homologs were not found in *Trichoplax*, suggesting the loss of this type of transporter in the placozoan lineage.

In summary, we infer that vGluTs diverged in the common ancestor of (Placozoa+Cnidaria + Bilateria), while vNuTs have a deeper, pre-metazoan ancestry.

## Discussion

### Locomotion, feeding, and integrative systems in Placozoa

Here, we observed spontaneous oscillations in the locomotion of *Trichoplax adhaerens*. We also revealed the induction and modulation of endogenous locomotory and feeding cycles by transmitter candidates such as enantiomers of glutamate and aspartate, GABA, glycine, and ATP. The administration of each of these molecules resulted in induction or inhibition of well-coordinated behavioral responses suggesting that these are endogenous signal molecules. They might represent the cohort of signaling molecules, enabling behavioral integration in the common metazoan ancestor (Moroz et al., 2021b).

Integrative systems in biology express themselves in global electrical, chemical, and mechanical oscillations on the scale of the entire brain or organism (Hanson, 2021). These low-frequency electrical oscillations are often spontaneous (not caused by external stimuli), ubiquitously found in neural systems from *Hydra* to humans, and are also shown for higher plants, fungi, and bacterial biofilms (Hanson, 2021). Spontaneous mechanical and chemical oscillations are well-known as growth and development regulation factors from hydrozoans to mammals (Tsiairis and Aulehla, 2016; Holmes et al., 2018; Marfenin and Dementyev, 2019).

In simpler animals like *Trichoplax*, analyses of mechanisms and integrative functions of such behavioral oscillations are important because placozoans lack chemical or electric synapses, gap junctions, pannexin, and connexin genes.

For example, the feeding cycle in placozoans includes coordinated changes in cilia beating, substrate adhesion, and body shape changes. Under the volume transmission hypothesis (Moroz et al., 2021b), we can expect the integrative function of established but dynamic chemical gradients (e.g., amino acids) in placozoans. Predicted chemical oscillations can be coupled to electrical signals and play a role in information processing (Romanova et al., 2020b). Mechanical integration is also possible as ultrafast contraction waves propagating across the placozoan body were observed (Armon et al., 2018).

Glutamate and ATP are among the most ancient signaling molecules in all life domains. They are abundant in cytoplasm, released to the extracellular medium upon cell rupture, and used as injury signals in a wide range of organisms, including plants and bacteria (Moroz et al., 2021b). GABA is a metabolite of glutamate pathways and is broadly utilized as a signaling molecule even in unicellular eukaryotes (Delmonte Corrado et al., 2002; Bucci et al., 2005). Here, we show that both pathways are involved in controlling feeding and locomotory patterns without overlapping functions, although the sources and cellular targets of glutamate/GABA release and action are currently unknown. The predicted sources could be primary extracellular such as products of extracellular digestion following its diffusion or selected uptake. However, specific glutamatergic or GABAergic cells might also exist. Such cells can selectively accumulate these molecules and release them endogenously or following selective stimuli. The co-existence of both scenarios is to be determined in future studies.

The genome of *Trichoplax adhaerens* encodes numerous components of predicted peptidergic, glutamatergic, GABAergic, purinergic (ATP), and nitric oxide-mediated transmission components (Srivastava et al., 2008; Moroz and Kohn, 2015; Moroz et al., 2020a,b, 2021b) but their spatial cellular distribution is also unknown. It was shown that secreted peptides could regulate some parts of the feeding cycle, some homologous to vertebrate endomorphins (Senatore et al., 2017; Varoqueaux et al., 2018) and peptidergic cells might also use certain amino acids as co-transmitters with canonical or novel regulatory release mechanisms. Some peptide-secreting cells are flask-shaped, have primary sensory cilia, and express secretory (exocytosis/presynaptic-like) components such as SNAP-25 and synaptobrevin (Smith et al., 2014).

We conclude that *Trichoplax* utilizes peptides (Senatore et al., 2017; Varoqueaux et al., 2018) and amino acids (our data) as endogenous signaling molecules. In the nervous systems of vertebrates and many invertebrates peptides exert slower effects *via* metabotropic receptors, while amino acids usually have faster postsynaptic actions mediated by ionotropic receptors (Greengard, 2001; Jing and Weiss, 2001; Nusbaum et al., 2001). This distinction is less evident in cnidarians and other invertebrates with a diverse set of peptide-gated ion channels of the DEG/ENAC family, which mediate fast signaling (Lingueglia et al., 1995; Grunder and Assmann, 2015; Schmidt et al., 2018). *Trichoplax* behavioral reactions are relatively slow, on the order of minutes, and ionotropic and metabotropic receptor-mediated effects are within the physiological range.

### Baseline description of *Trichoplax* behavior

There are several descriptions of *Trichoplax* behavior in the existing literature (Okshtein, 1987; Seravin, 1989; Ueda et al., 1999; Heyland et al., 2014; Smith et al., 2015; Senatore et al., 2017; Fortunato and Aktipis, 2019; Smith et al., 2019). However, all papers before the 1990s used manual drawings or short photo sequences based on observations of a few animals and lacked statistical analysis. More recent articles (Smith and Mayorova, 2019) focused on shorter and smaller scales—several mm, compared to the 52 mm dish in our work. Only one paper reported video recording and statistical analysis of placozoan locomotion on a timescale of hours and spatial scale of over 10 mm (Ueda et al., 1999), but it does not present any tracks. It is based on an unknown (presumably small) number of observed animals.

We have recorded and digitized tracks for over 2,500 animal hours of normal placozoan behavior, testing at least 50 animals for each type of behavior and using long record times of up to 24 h. This is the most comprehensive analysis of normal placozoan behaviors performed so far (see also a recent preprint by Zhong et al. (2022) about a very detailed analysis of *Trichoplax* thermotaxis). Also, we made an effort to standardize animal conditions before testing by using only animals from the late exponential phase of culture growth. This approach helped to mitigate the poor reproducibility of placozoan behavior.

These efforts allowed us to observe the long-term dynamics of endogenous placozoan locomotion patterns: gradual acceleration of locomotion and its long-term oscillations. Gradual acceleration was observed when video recording was longer than 2 h and must be considered in all prolonged experiments with *Trichoplax*. These datasets also provide unique opportunities to study integrative systems of placozoans underlying long-term modulation of behaviors in the simplest free-living animals.

### Locomotory and feeding oscillations and the integrative systems of Placozoa

*Trichoplax*, an animal without neurons, synapses, and muscles, exhibits the complex feeding cycle—a coordinated and stereotyped sequence of patterns leading to changes in cilia beating, body shape, cell adhesion, and secretion (Smith et al., 2015). The feeding cycle is activated by external stimuli (food), but it might also occur spontaneously due to endogenous center pattern generator activity phenomenologically similar to bilaterians and cnidarians. We also report endogenous oscillations in *Trichoplax*’s locomotory rhythm for directional motion and rotations.

High [Mg^2+^] or low [Ca^2+^] have abolished endogenous locomotory and feeding oscillations, suggesting the requirement for intercellular chemical communications for these behaviors. A high magnesium concentration causes stable hyperpolarization of cell membranes and suppresses action potentials. Low [Ca^2+^] also decreases the evolutionarily conserved machinery of calcium-dependent exocytosis. These observations are consistent with the hypothesis of volume transmission as the ancestral integrative systems using electrochemical vesicular secretion (Moroz et al., 2021b).

### Modulation of rhythmic patterns of the *Trichoplax* behavior by amino acid transmitters and ATP

L-glutamate is a versatile signal molecule in various organisms – from prokaryotes to bilaterians (Moroz et al., 2021a). D-aspartate is a co-agonist of NMDA-type glutamate receptors and was found in *Trichoplax* in sub-millimolar concentrations (Moroz et al., 2020b). D-glutamate also induced action potentials and muscle contraction in ctenophores (Moroz et al., 2014).

We have demonstrated that glutamate, aspartate, glycine, and GABA profoundly affect the locomotory and feeding patterns of the *Trichoplax*. L-glutamate (and, to less extent, aspartate isomers and D-glutamate) turn on the feeding-like behavior sequence even in the absence of food. Glycine and GABA have more subtle modulating effects. Glycine suppresses feeding on algae and increases curvature tracks on clean dishes. GABA suppresses feeding and locomotory rhythms and acts on orders for several hours.

Thus, GABA and glycine might act as antagonists of glutamate in regulating *Trichoplax* feeding behaviors. In mammalian CNS, glutamate is a predominant excitatory transmitter, while GABA and glycine—are primary inhibitory transmitters. The functional ‘antagonism’ of GABA and glutamate can be an ancient trait related to their metabolic relationships (Moroz et al., 2021a). GABA is synthesized from glutamate by glutamate decarboxylase. When the time-limited response to glutamate is desired, an antagonistic response to GABA is the simplest way to coordinate the overall outcome. The concerted action of these two transmitters forms a feed-forward loop (Alon, 2007).

L-Glutamate is a universal animal ‘food’ signal (Torii et al., 2013; Moroz et al., 2021a). It is not surprising that in *Trichoplax,* glutamate also invokes components of feeding-like behavior. It is interesting that a high concentration of L-glutamate (3 mM) caused irreversible body expansion in addition to other components of the feeding cycles (ciliary beating arrest, adhesion, and deadhesion). Enantiomers of aspartate do not show this effect in all tested concentrations. Such a situation may point to the dual role of L-glutamate in the placozoan feeding sequence: (1) initiation of feeding sequence, also caused by aspartate, and (2) regulation of body expansion and contraction, more associated with L-glutamate.

### Comparison with the sponge integrative systems

Sponges (phylum Porifera) are sedentary filtrators. Their coordinated locomotory responses are limited to contractions of the osculum (water canal system opening), rhythmic contractions of the entire canal system, and “sneezing”—biphasic expansion and contraction of the whole body to remove the unwanted particles from canals (Leys, 2015). Most sponges are challenging to maintain in the lab, and most experimental data come from freshwater *Ephydatia muelleri* and marine *Tethya wilhelma*. *Tethya* responds with “sneeze” to glutamate, GABA, glycine, acetylcholine, serotonin, dopamine, cyclic AMP, caffeine, and nicotine (Ellwanger and Nickel, 2006; Ellwanger et al., 2007). In *Ephydatia*, glutamate stimulates “sneeze,” while 0.1 mM GABA suppresses “sneeze” responses both to glutamate and mineral particles (Elliott and Leys, 2010). Mammalian mGluR antagonists AP3 and kynurenic acid inhibit ‘sneeze’ of *Ephydatia* in response to glutamate and mineral particles (Elliott and Leys, 2010), suggesting the involvement of mGluRs in the coordination of this reaction.

The role of action potentials and other electric processes in sponge coordination is unclear. An early study by Prosser (1967) has shown that contractions in marine sponges are regular in high extracellular potassium (100 mM) and even increase in amplitude in seawater with a complete exchange of sodium to potassium (450 mM). These high [K^+^] concentrations cause stable membrane depolarization and complete blockade of action potentials. Therefore, desmosponges seem to coordinate body contraction without classical Na-dependent action potentials or have action potentials based on another set of ions.

In summary, we emphasize that glutamate causes coordinated behaviors in both sponges and placozoans. Effects of GABA are likely antagonistic to glutamate in *Trichoplax* and *Ephydatia* but synergistic in *Tethya.* Our data on the effects of high [Mg^2+^] and low [Ca^2+^] are compatible with the role of calcium-dependent exocytosis of signal molecules in integrating *Trichoplax* behaviors. However, the role of the Ca-dependent secretion of signal molecules in the coordination of sponge behavior is unknown.

### Summary and perspectives

Our experiments provide initial information on the roles of glutamate, GABA, glycine, and ATP in integrating placozoan behaviors. This situation might represent the ancestral models of chemical integration, *via* volume transmission, in early metazoans without canonical neurons and synapses.

However, future experiments must be conducted by deciphering cellular bases of multiple behaviors to demonstrate the specific roles of these amino acids (and other signal molecules) in Placozoa conclusively. The first priority is the unbiased identification of endogenous sources of glutamate, GABA, glycine, and ATP: the identification of specific cells and release mechanisms. In other words, we need to know the localization, quantitative secretion of these molecules, and the distribution of such hypothetical chemoconnectomes in the simplest free-living metazoans (Moroz et al., 2021b). The strategy can employ conventional approaches (e.g., single-cell RNA-seq, *in situ* hybridization to putative glutamate vesicular transporter mRNAs, immunocytochemistry, etc.). However, the predicted dynamic nature of numerous chemical gradients requires innovative components for real-time physiological measurements of intercellular communications in placozoans, complemented with microchemical quantitative assays.

## Data availability statement

The datasets presented in this study can be found in online repositories. The names of the repository/repositories and accession number(s) can be found in the article/Supplementary material.

## Author contributions

MN, DR, and LM designed the study. MN and SB performed behavioral experiments, digitized animal tracks, and performed analyses of behavioral data. MN, LM, and SB identified genes encoding receptors and transporters and made phylogenetic trees. DR, MN, and LM made the figures and videos. MN, DR, and LM wrote the manuscript. All authors contributed to the article and approved the submitted version.

## Funding

MN and SB were supported by the Russian Science Foundation grant (22-24-00566). LM was supported by HFSP, NSF, and NIH grants as well as private donors through UF Foundation.

## Conflict of interest

The authors declare that the research was conducted in the absence of any commercial or financial relationships that could be construed as a potential conflict of interest.

## Publisher’s note

All claims expressed in this article are solely those of the authors and do not necessarily represent those of their affiliated organizations, or those of the publisher, the editors and the reviewers. Any product that may be evaluated in this article, or claim that may be made by its manufacturer, is not guaranteed or endorsed by the publisher.

## Footnotes

^1^ http://avidemux.sourceforge.net/

^2^ https://mafft.cbrc.jp/alignment/server/

## Supplementary material

Supplemental video S1 Normal locomotion of *Trichoplax adhaerens* on the clean glass. 10X magnification, 300X time-lapse. Seven animals are crawling freely. Tracks of four animals are shown in pink, blue, green, and yellow.

Supplementary video S2 Normal feeding of *Trichoplax *on cyanobacterial biofilm. 200X magnification, 300X time-lapse. One can see 6 feeding cycles in 42 minutes in real time.

Supplementary video S3 Normal locomotion of *Trichoplax* adhaerens, same specimen whose locomotion was plotted in Figures 2C,D. 10X magnification, 300X time-lapse. The animal track is shown in pink.

Supplementary video S4 Acceleration of normal locomotion over time. 10X magnification, 300X time-lapse. Animals glide on clean glass (first half of video) and after 12 hours.

Supplementary video S5 Effect of 200 mM MgCl_2_ on locomotion. 10X magnification, 600X time-lapse. In control, animals crawl around. After adding MgCl2, animals gradually (~10 min) cease directed movements and stay in one place but continue to change shape.

Supplementary video S6 Effects of low Ca^2+^ concentration (<1.5 mM) on locomotion. 10X magnification, 600X time-lapse. In control, animals crawl around in small drops of seawater. After adding Ca-free seawater, animals immediately (<1min) cease directed movements and stay in one place but continue to change shape.

Supplementary video S7 Feeding-like cycles of *Trichoplax* induced by 0.1 mM L-glutamate in the absence of food. 80X magnification, 300X time-lapse. In control, the animal moves around on the glass, and a single feeding-like cycle is observed. After the addition of glutamate, a succession of four feeding-like cycles is induced.

Supplementary video S8 Effect of 3 mM L-glutamate: animal permanently expanded. One can see that upon the addition of 3mM L-Glu animal expands like at the beginning of the feeding cycle but never contracts back. 80X magnification, 300X time-lapse.

Supplementary video S9 Feeding suppression by 0.3 mM glycine. In the first half of the video, animals feed on cyanobacterial biofilm. After the addition of glycine, most of them stopped feeding and began to move around. 10X magnification, 600X time-lapse.

Supplemental video S10 Feeding suppression by 0.3 mM GABA. In control, animals feed on cyanobacterial biofilm. After 6 hours in 0.3 mM GABA, they quickly move around but barely stop to eat. 10X magnification, 600X time-lapse.

Supplementary video S11 Effects of 0.3 mM of GABA on locomotion. 10X magnification, 600X time-lapse. In control, animals crawl around. After 6 hours in 0.3 mM GABA, animals wobbled most of the time in one place and rarely changed position.

Supplementary video S12 Effects of 0.3 mM of glycine on locomotion. 10X magnification, 600X time-lapse. Tracks of four example animals are shown in pink (control) and blue (after glycine addition). After the addition of 0.3 mM glycine, animal tracks become more convoluted.

Supplementary table S1 Sequences and accession numbers of proteins used for phylogenetic trees.

Supplementary table S2 Source databases for protein sequences that were used in this paper.

Supplementary figure 1 Effects of GABA on the locomotion of *Trichoplax* on a conditioned dish. **(A)** Animal locomotion on a clean dish is increased over time in control but slightly decreases during incubation with 0.3 or 1 mM GABA. Asterisks denote a significant difference in speed from the control (Welch *t*-test, *** - *p* < 0.001). N animals = 98, 50, 74, 29, 46, 69, 35, 44, and 72, respectively. **(B)**. Track straightness on a clean dish does not change over time in control but decreases during incubation with GABA (tracks become more convoluted). N animals are the same as in A.

Supplementary figure 2 Comparison of feeding dynamics on green algae *Tetraselmis* and cyanobacteria *Oscillatoria*. After 12 hours, feeding on cyanobacteria is less active than on green algae, and the standard deviation of feeding activity is higher on cyanobacteria. N animals = 40 for green algae and 48 for cyanobacteria.

Supplementary figure 3 Bayesian phylogenetic tree of ionotropic glutamate receptors. Numbers denote bayesian posterior probability near nodes. Analysis was run for 1.42 million generations, and convergence was checked with Tracer. Sequences were aligned using E-INS-i algorithm implemented in MAFFT online. Alignment consisted of 4071 positions, 1464 of them parsimony-informative.

Supplementary figure 4 Maximum Likelihood phylogenetic tree of metabotropic glutamate and related receptors. Numbers denote ultrafast bootstrap support near nodes. Sequences were aligned using E-INS-i algorithm implemented in MAFFT online. Alignment consisted of 4071 positions, 1464 of them parsimony-informative.

Supplementary figure 5 Maximum Likelihood phylogenetic tree of metabotropic GABA receptors. Numbers denote ultrafast bootstrap support near nodes. Sequences were aligned using E-INS-i algorithm implemented in MAFFT online. Alignment consisted of 5312 positions, 1376 of them parsimony-informative.

Supplementary figure 6 Bayesian phylogenetic tree of vesicular glutamate and related transporters (SLC17 family). Numbers denote bayesian posterior probability near nodes. Analysis was run for 10 million generations, and convergence was checked with Tracer. Sequences were aligned using E-INS-i algorithm implemented in MAFFT online. Alignment consisted of 1036 positions, 603 of them parsimony-informative.

## Abbreviations

ATP: adenosine triphosphate
ASW: artificial seawater
GABA: gamma-aminobutyric acid
GABA-BR: gamma-aminobutyric acid receptor type B
mGluR: metabotropic glutamate receptor
iGluR: ionotropic glutamate receptor
CaSR: extracellular calcium-sensing receptor
vGluT: vesicular glutamate transporter
vNuT: vesicular nucleotide transporter
