# Supplementary material for: Amino acids integrate behaviors in nerveless placozoans
Source: Front Neurosci. 2023 Apr 13;17:1125624. doi: 10.3389/fnins.2023.1125624 (PMC10133484; doi:10.3389/fnins.2023.1125624)
Supplement: Supplementary figure 1 — Effects of GABA on the locomotion of Trichoplax on a conditioned dish. (A) Animal locomotion on a clean dish is increased over time in control but slightly decreases during incubation with 0.3 or 1 mM GABA. Asterisks denote a significant difference in speed from the control (Welch t-test, *** - p < 0.001). N animals = 98, 50, 74, 29, 46, 69, 35, 44, and 72, respectively. (B). Track straightness on a clean dish does not change over time in control but decreases during incubation with GABA (tracks become more convoluted). N animals are the same as in A. [file Data_Sheet_3.pdf]

Supplemental Figures:

Glutamate, aspartate, GABA, glycine, and ATP integrate locomotion and feeding behaviors in placozoans

Mikhail A. Nikitin, Darja Y. Romanova, Simkha I. Borman, Leonid L. Moroz

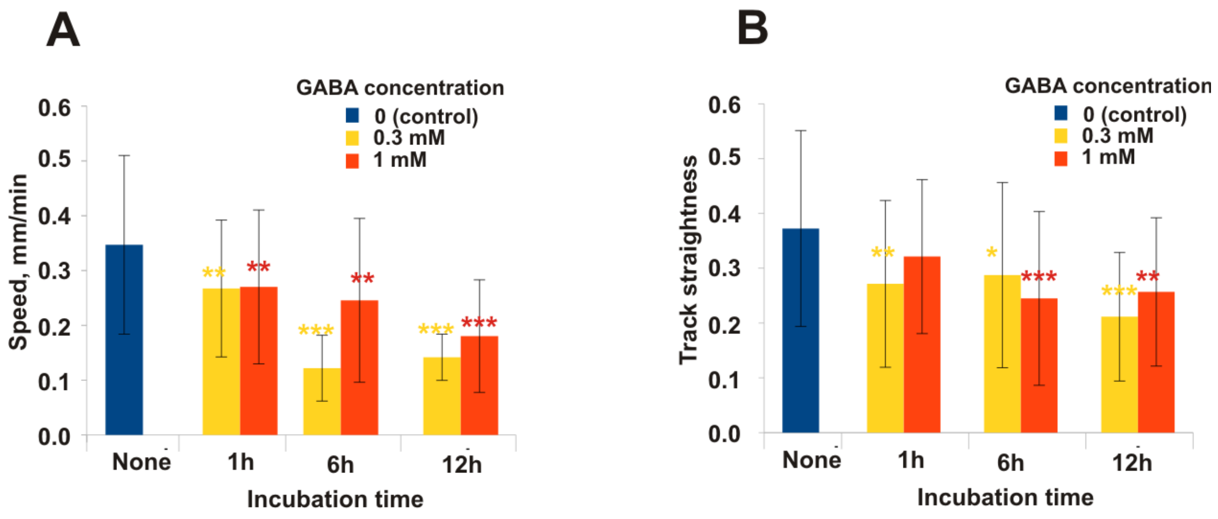

Supplemental Figure 1. Effects of GABA on the locomotion of *Trichoplax* on a conditioned dish. **(A)** Animal locomotion on a clean dish is increased over time in control but slightly decreases during incubation with 0.3 or 1 mM GABA. Asterisks denote a significant difference in speed from the control (Welch t-test, *** - p < 0.001). N animals = 98, 50, 74, 29, 46, 69, 35, 44, and 72, respectively. **(B)**. Track straightness on a clean dish does not change over time in control but decreases during incubation with GABA (tracks become more convoluted). N animals are the same as in A.

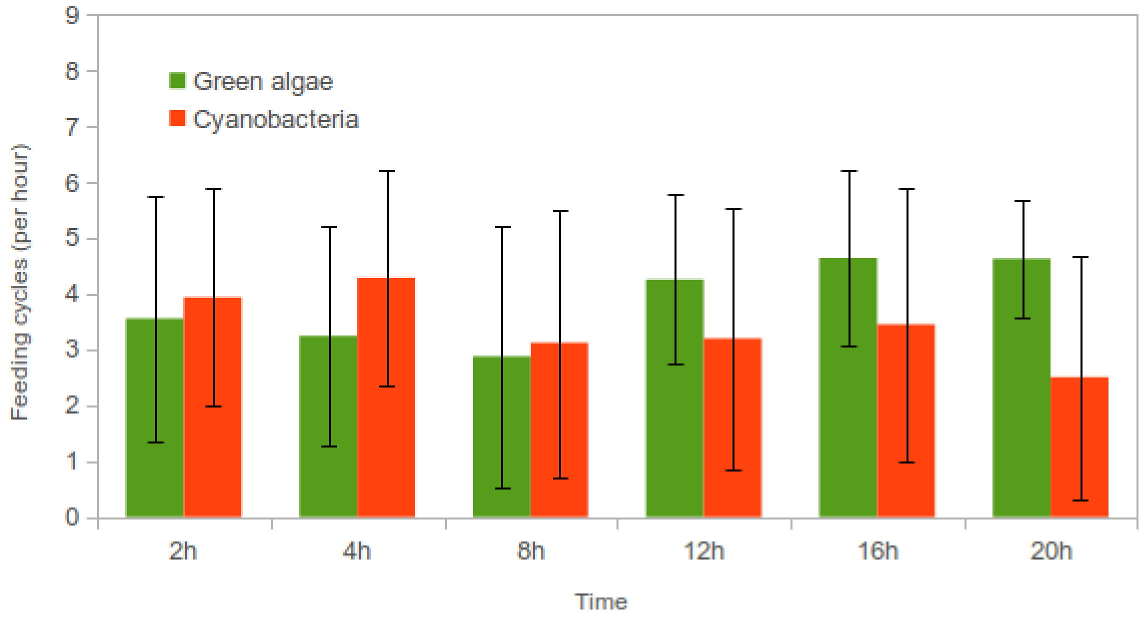

Supplemental figure S2. Comparison of feeding dynamics on green algae *Tetraselmis* and cyanobacteria *Oscillatoria*. After 12 hours, feeding on cyanobacteria is less active than on green algae, and the standard deviation of feeding activity is higher on cyanobacteria. N animals = 40 for green algae and 48 for cyanobacteria.

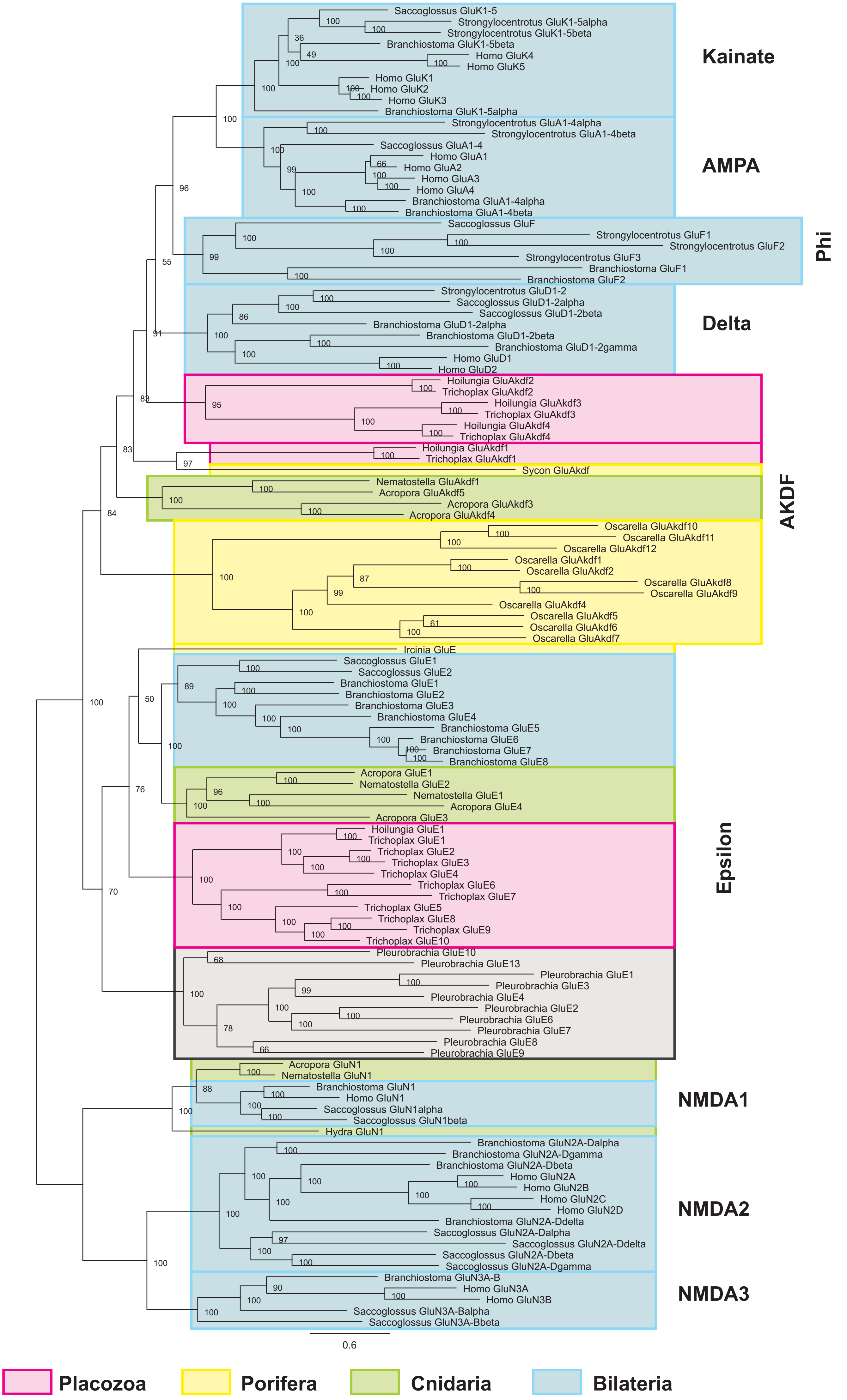

Saccoglossus GluK1-5
Strongylocentrotus GluK1-5alpha
Strongylocentrotus GluK1-5beta
Branchiostoma GluK1-5beta
Homo GluK4
Homo GluK5
Homo GluK1
Homo GluK2
Homo GluK3
Branchiostoma GluK1-5alpha
Kainate
Strongylocentrotus GluA1-4alpha
Strongylocentrotus GluA1-4beta
Saccoglossus GluA1-4
Homo GluA1
Homo GluA2
Homo GluA3
Homo GluA4
Branchiostoma GluA1-4alpha
Branchiostoma GluA1-4beta
AMPA
Saccoglossus GluF
Strongylocentrotus GluF1
Strongylocentrotus GluF2
Strongylocentrotus GluF3
Branchiostoma GluF1
Branchiostoma GluF2
Phi
Strongylocentrotus GluD1-2
Saccoglossus GluD1-2alpha
Saccoglossus GluD1-2beta
Branchiostoma GluD1-2alpha
Branchiostoma GluD1-2beta
Branchiostoma GluD1-2gamma
Homo GluD1
Homo GluD2
Delta
Hoilungia GluAkdf2
Trichoplax GluAkdf2
Hoilungia GluAkdf3
Trichoplax GluAkdf3
Hoilungia GluAkdf4
Trichoplax GluAkdf4
Hoilungia GluAkdf1
Trichoplax GluAkdf1
Sycon GluAkdf
Nematostella GluAkdf1
Acropora GluAkdf5
Acropora GluAkdf3
Acropora GluAkdf4
Oscarella GluAkdf10
Oscarella GluAkdf11
Oscarella GluAkdf12
Oscarella GluAkdf1
Oscarella GluAkdf2
Oscarella GluAkdf8
Oscarella GluAkdf9
Oscarella GluAkdf4
Oscarella GluAkdf5
Oscarella GluAkdf6
Oscarella GluAkdf7
AKDF
Ircinia GluE
Saccoglossus GluE1
Saccoglossus GluE2
Branchiostoma GluE1
Branchiostoma GluE2
Branchiostoma GluE3
Branchiostoma GluE4
Branchiostoma GluE5
Branchiostoma GluE6
Branchiostoma GluE7
Branchiostoma GluE8
Acropora GluE1
Nematostella GluE2
Nematostella GluE1
Acropora GluE4
Acropora GluE3
Hoilungia GluE1
Trichoplax GluE1
Trichoplax GluE2
Trichoplax GluE3
Trichoplax GluE4
Trichoplax GluE6
Trichoplax GluE7
Trichoplax GluE5
Trichoplax GluE8
Trichoplax GluE9
Trichoplax GluE10
Pleurobrachia GluE10
Pleurobrachia GluE13
Pleurobrachia GluE1
Pleurobrachia GluE3
Pleurobrachia GluE4
Pleurobrachia GluE2
Pleurobrachia GluE6
Pleurobrachia GluE7
Pleurobrachia GluE8
Pleurobrachia GluE9
Epsilon
Acropora GluN1
Nematostella GluN1
Branchiostoma GluN1
Homo GluN1
Saccoglossus GluN1alpha
Saccoglossus GluN1beta
Hydra GluN1
NMDA1
Branchiostoma GluN2A-Dalpha
Branchiostoma GluN2A-Dgamma
Branchiostoma GluN2A-Dbeta
Homo GluN2A
Homo GluN2B
Homo GluN2C
Homo GluN2D
Branchiostoma GluN2A-Ddelta
Saccoglossus GluN2A-Dalpha
Saccoglossus GluN2A-Ddelta
Saccoglossus GluN2A-Dbeta
Saccoglossus GluN2A-Dgamma
NMDA2
Branchiostoma GluN3A-B
Homo GluN3A
Homo GluN3B
Saccoglossus GluN3A-Balpha
Saccoglossus GluN3A-Bbeta
NMDA3
0.6
Placozoa
Porifera
Cnidaria
Bilateria

Supplemental figure S3. Bayesian phylogenetic tree of ionotropic glutamate receptors. Numbers denote bayesian posterior probability near nodes. Analysis was run for 1.42 million generations, and convergence was checked with Tracer. Sequences were aligned using E-INS-i algorithm implemented in MAFFT online. Alignment consisted of 2472 positions, 1348 of them parsimony-informative.

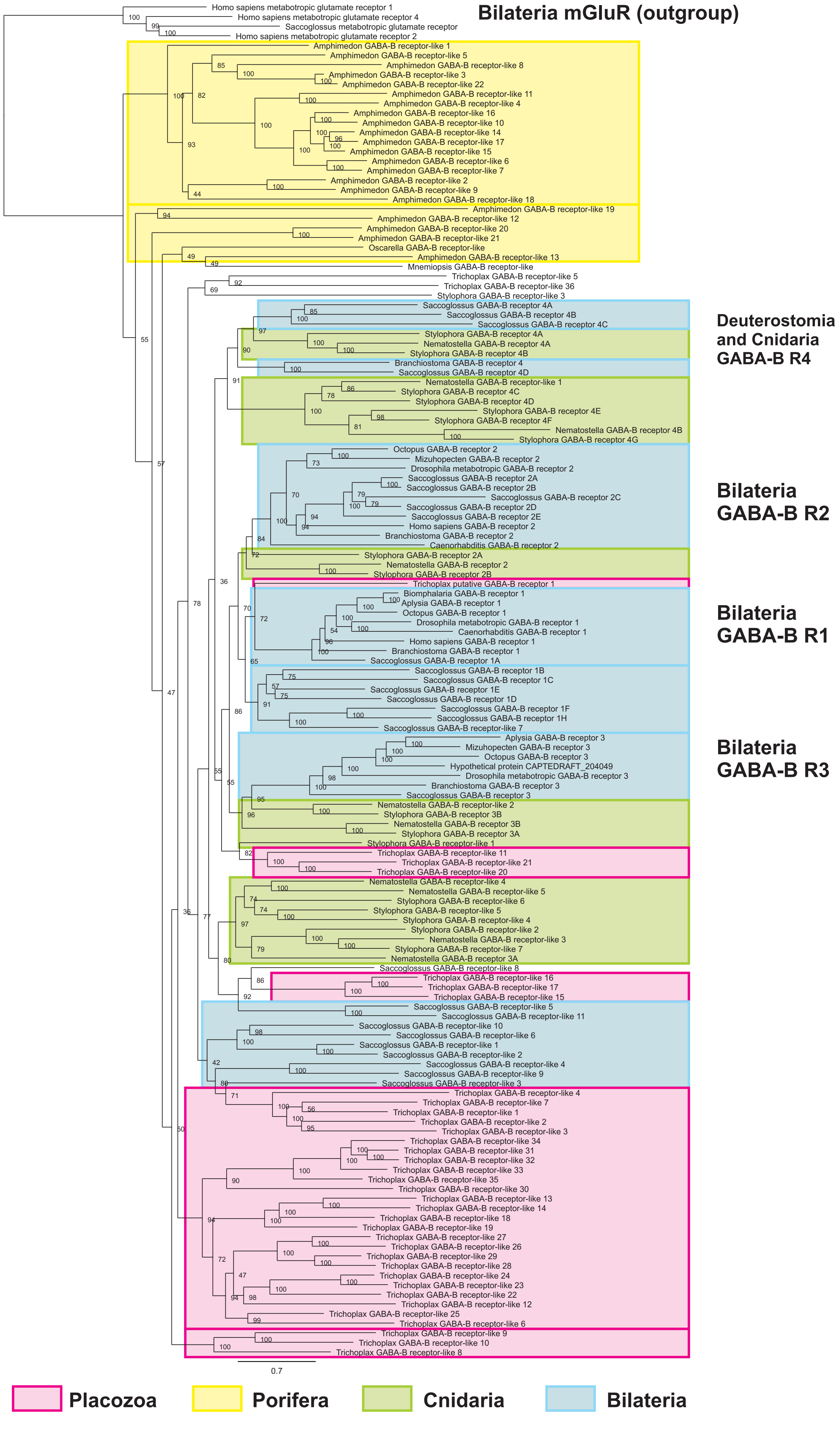
Bilateria mGluR (outgroup)
Homo sapiens metabotropic glutamate receptor 1
Homo sapiens metabotropic glutamate receptor 4
Saccoglossus metabotropic glutamate receptor
Homo sapiens metabotropic glutamate receptor 2
Amphimedon GABA-B receptor-like 1
Amphimedon GABA-B receptor-like 5
Amphimedon GABA-B receptor-like 8
Amphimedon GABA-B receptor-like 3
Amphimedon GABA-B receptor-like 22
Amphimedon GABA-B receptor-like 11
Amphimedon GABA-B receptor-like 4
Amphimedon GABA-B receptor-like 16
Amphimedon GABA-B receptor-like 10
Amphimedon GABA-B receptor-like 14
Amphimedon GABA-B receptor-like 17
Amphimedon GABA-B receptor-like 15
Amphimedon GABA-B receptor-like 6
Amphimedon GABA-B receptor-like 7
Amphimedon GABA-B receptor-like 2
Amphimedon GABA-B receptor-like 9
Amphimedon GABA-B receptor-like 18
Amphimedon GABA-B receptor-like 19
Amphimedon GABA-B receptor-like 12
Amphimedon GABA-B receptor-like 20
Amphimedon GABA-B receptor-like 21
Oscarella GABA-B receptor-like
Amphimedon GABA-B receptor-like 13
Mnemiopsis GABA-B receptor-like
Trichoplax GABA-B receptor-like 5
Trichoplax GABA-B receptor-like 36
Stylophora GABA-B receptor-like 3
Deuterostomia and Cnidaria GABA-B R4
Saccoglossus GABA-B receptor 4A
Saccoglossus GABA-B receptor 4B
Saccoglossus GABA-B receptor 4C
Stylophora GABA-B receptor 4A
Nematostella GABA-B receptor 4A
Stylophora GABA-B receptor 4B
Branchiostoma GABA-B receptor 4
Saccoglossus GABA-B receptor 4D
Nematostella GABA-B receptor-like 1
Stylophora GABA-B receptor 4C
Stylophora GABA-B receptor 4D
Stylophora GABA-B receptor 4E
Stylophora GABA-B receptor 4F
Nematostella GABA-B receptor 4B
Stylophora GABA-B receptor 4G
Bilateria GABA-B R2
Octopus GABA-B receptor 2
Mizuhopecten GABA-B receptor 2
Drosophila metabotropic GABA-B receptor 2
Saccoglossus GABA-B receptor 2A
Saccoglossus GABA-B receptor 2B
Saccoglossus GABA-B receptor 2C
Saccoglossus GABA-B receptor 2D
Saccoglossus GABA-B receptor 2E
Homo sapiens GABA-B receptor 2
Branchiostoma GABA-B receptor 2
Caenorhabditis GABA-B receptor 2
Stylophora GABA-B receptor 2A
Nematostella GABA-B receptor 2
Stylophora GABA-B receptor 2B
Bilateria GABA-B R1
Trichoplax putative GABA-B receptor 1
Biomphalaria GABA-B receptor 1
Aplysia GABA-B receptor 1
Octopus GABA-B receptor 1
Drosophila metabotropic GABA-B receptor 1
Caenorhabditis GABA-B receptor 1
Homo sapiens GABA-B receptor 1
Branchiostoma GABA-B receptor 1
Saccoglossus GABA-B receptor 1A
Saccoglossus GABA-B receptor 1B
Saccoglossus GABA-B receptor 1C
Saccoglossus GABA-B receptor 1E
Saccoglossus GABA-B receptor 1D
Saccoglossus GABA-B receptor 1F
Saccoglossus GABA-B receptor 1H
Saccoglossus GABA-B receptor-like 7
Bilateria GABA-B R3
Aplysia GABA-B receptor 3
Mizuhopecten GABA-B receptor 3
Octopus GABA-B receptor 3
Hypothetical protein CAPTEDRAFT_204049
Drosophila metabotropic GABA-B receptor 3
Branchiostoma GABA-B receptor 3
Saccoglossus GABA-B receptor 3
Nematostella GABA-B receptor-like 2
Stylophora GABA-B receptor 3B
Nematostella GABA-B receptor 3B
Stylophora GABA-B receptor 3A
Stylophora GABA-B receptor-like 1
Trichoplax GABA-B receptor-like 11
Trichoplax GABA-B receptor-like 21
Trichoplax GABA-B receptor-like 20
Nematostella GABA-B receptor-like 4
Nematostella GABA-B receptor-like 5
Stylophora GABA-B receptor-like 6
Stylophora GABA-B receptor-like 5
Stylophora GABA-B receptor-like 4
Stylophora GABA-B receptor-like 2
Nematostella GABA-B receptor-like 3
Stylophora GABA-B receptor-like 7
Nematostella GABA-B receptor 3A
Saccoglossus GABA-B receptor-like 8
Trichoplax GABA-B receptor-like 16
Trichoplax GABA-B receptor-like 17
Trichoplax GABA-B receptor-like 15
Saccoglossus GABA-B receptor-like 5
Saccoglossus GABA-B receptor-like 11
Saccoglossus GABA-B receptor-like 10
Saccoglossus GABA-B receptor-like 6
Saccoglossus GABA-B receptor-like 1
Saccoglossus GABA-B receptor-like 2
Saccoglossus GABA-B receptor-like 4
Saccoglossus GABA-B receptor-like 9
Saccoglossus GABA-B receptor-like 3
Trichoplax GABA-B receptor-like 4
Trichoplax GABA-B receptor-like 7
Trichoplax GABA-B receptor-like 1
Trichoplax GABA-B receptor-like 2
Trichoplax GABA-B receptor-like 3
Trichoplax GABA-B receptor-like 34
Trichoplax GABA-B receptor-like 31
Trichoplax GABA-B receptor-like 32
Trichoplax GABA-B receptor-like 33
Trichoplax GABA-B receptor-like 35
Trichoplax GABA-B receptor-like 30
Trichoplax GABA-B receptor-like 13
Trichoplax GABA-B receptor-like 14
Trichoplax GABA-B receptor-like 18
Trichoplax GABA-B receptor-like 19
Trichoplax GABA-B receptor-like 27
Trichoplax GABA-B receptor-like 26
Trichoplax GABA-B receptor-like 29
Trichoplax GABA-B receptor-like 28
Trichoplax GABA-B receptor-like 24
Trichoplax GABA-B receptor-like 23
Trichoplax GABA-B receptor-like 22
Trichoplax GABA-B receptor-like 12
Trichoplax GABA-B receptor-like 25
Trichoplax GABA-B receptor-like 6
Trichoplax GABA-B receptor-like 9
Trichoplax GABA-B receptor-like 10
Trichoplax GABA-B receptor-like 8
0.7
Placozoa
Porifera
Cnidaria
Bilateria

Supplemental figure S4. Maximum Likelihood phylogenetic tree of metabotropic glutamate and related receptors. Numbers denote ultrafast bootstrap support near nodes. Sequences were aligned using E-INS-i algorithm implemented in MAFFT online. Alignment consisted of 4071 positions, 1464 of them parsimony-informative.

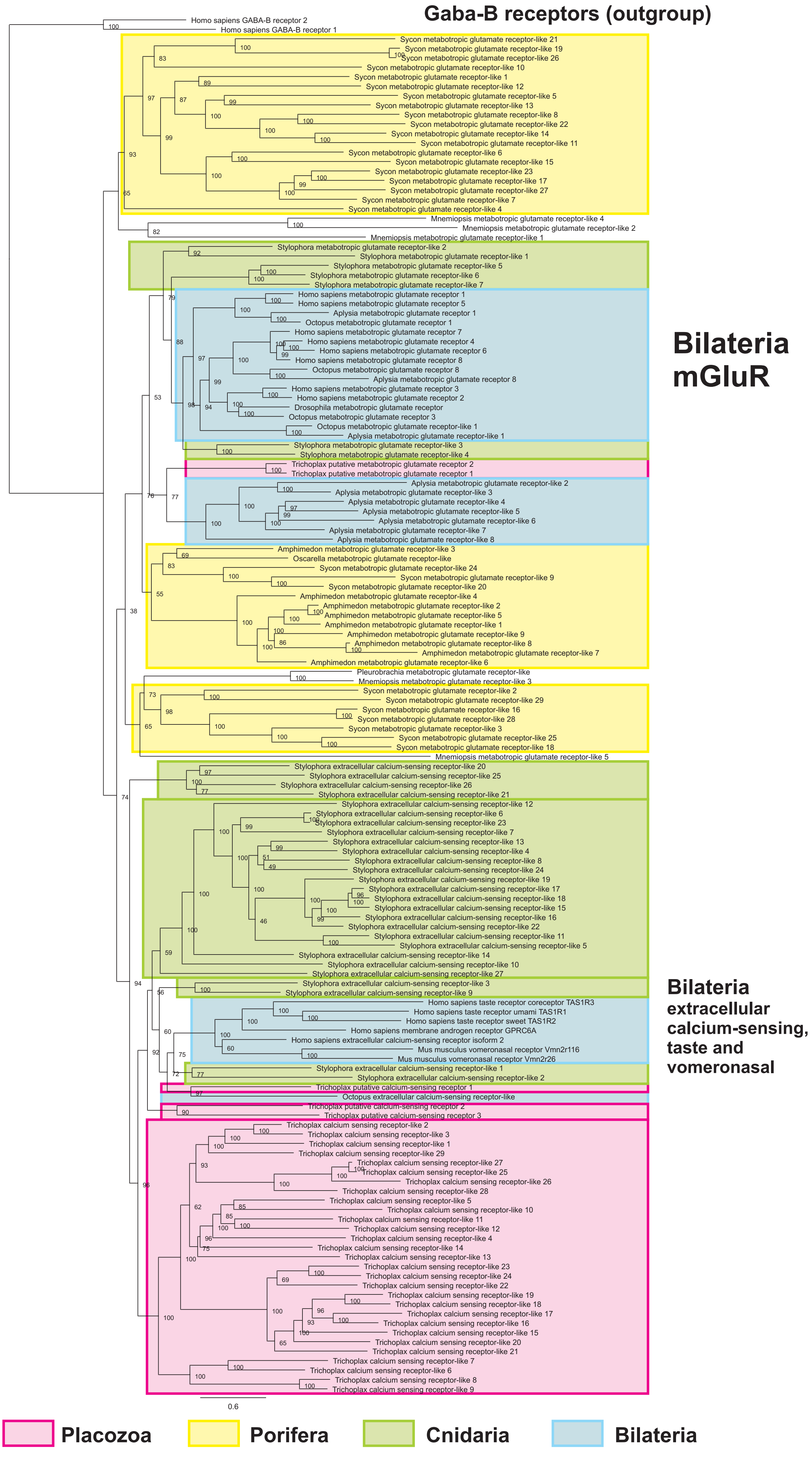

Gaba-B receptors (outgroup)
Homo sapiens GABA-B receptor 2
Homo sapiens GABA-B receptor 1
Sycon metabotropic glutamate receptor-like 21
Sycon metabotropic glutamate receptor-like 19
Sycon metabotropic glutamate receptor-like 26
Sycon metabotropic glutamate receptor-like 10
Sycon metabotropic glutamate receptor-like 1
Sycon metabotropic glutamate receptor-like 12
Sycon metabotropic glutamate receptor-like 5
Sycon metabotropic glutamate receptor-like 13
Sycon metabotropic glutamate receptor-like 8
Sycon metabotropic glutamate receptor-like 22
Sycon metabotropic glutamate receptor-like 14
Sycon metabotropic glutamate receptor-like 11
Sycon metabotropic glutamate receptor-like 6
Sycon metabotropic glutamate receptor-like 15
Sycon metabotropic glutamate receptor-like 23
Sycon metabotropic glutamate receptor-like 17
Sycon metabotropic glutamate receptor-like 27
Sycon metabotropic glutamate receptor-like 7
Sycon metabotropic glutamate receptor-like 4
Mnemiopsis metabotropic glutamate receptor-like 4
Mnemiopsis metabotropic glutamate receptor-like 2
Mnemiopsis metabotropic glutamate receptor-like 1
Stylophora metabotropic glutamate receptor-like 2
Stylophora metabotropic glutamate receptor-like 1
Stylophora metabotropic glutamate receptor-like 5
Stylophora metabotropic glutamate receptor-like 6
Stylophora metabotropic glutamate receptor-like 7
Homo sapiens metabotropic glutamate receptor 1
Homo sapiens metabotropic glutamate receptor 5
Aplysia metabotropic glutamate receptor 1
Octopus metabotropic glutamate receptor 1
Homo sapiens metabotropic glutamate receptor 7
Homo sapiens metabotropic glutamate receptor 4
Homo sapiens metabotropic glutamate receptor 6
Homo sapiens metabotropic glutamate receptor 8
Octopus metabotropic glutamate receptor 8
Aplysia metabotropic glutamate receptor 8
Homo sapiens metabotropic glutamate receptor 3
Homo sapiens metabotropic glutamate receptor 2
Drosophila metabotropic glutamate receptor
Octopus metabotropic glutamate receptor 3
Octopus metabotropic glutamate receptor-like 1
Aplysia metabotropic glutamate receptor-like 1
Stylophora metabotropic glutamate receptor-like 3
Stylophora metabotropic glutamate receptor-like 4
Trichoplax putative metabotropic glutamate receptor 2
Trichoplax putative metabotropic glutamate receptor 1
Aplysia metabotropic glutamate receptor-like 2
Aplysia metabotropic glutamate receptor-like 3
Aplysia metabotropic glutamate receptor-like 4
Aplysia metabotropic glutamate receptor-like 5
Aplysia metabotropic glutamate receptor-like 6
Aplysia metabotropic glutamate receptor-like 7
Aplysia metabotropic glutamate receptor-like 8
Amphimedon metabotropic glutamate receptor-like 3
Oscarella metabotropic glutamate receptor-like
Sycon metabotropic glutamate receptor-like 24
Sycon metabotropic glutamate receptor-like 9
Sycon metabotropic glutamate receptor-like 20
Amphimedon metabotropic glutamate receptor-like 4
Amphimedon metabotropic glutamate receptor-like 2
Amphimedon metabotropic glutamate receptor-like 5
Amphimedon metabotropic glutamate receptor-like 1
Amphimedon metabotropic glutamate receptor-like 9
Amphimedon metabotropic glutamate receptor-like 8
Amphimedon metabotropic glutamate receptor-like 7
Amphimedon metabotropic glutamate receptor-like 6
Pleurobrachia metabotropic glutamate receptor-like
Mnemiopsis metabotropic glutamate receptor-like 3
Sycon metabotropic glutamate receptor-like 2
Sycon metabotropic glutamate receptor-like 29
Sycon metabotropic glutamate receptor-like 16
Sycon metabotropic glutamate receptor-like 28
Sycon metabotropic glutamate receptor-like 3
Sycon metabotropic glutamate receptor-like 25
Sycon metabotropic glutamate receptor-like 18
Mnemiopsis metabotropic glutamate receptor-like 5
Stylophora extracellular calcium-sensing receptor-like 20
Stylophora extracellular calcium-sensing receptor-like 25
Stylophora extracellular calcium-sensing receptor-like 26
Stylophora extracellular calcium-sensing receptor-like 21
Stylophora extracellular calcium-sensing receptor-like 12
Stylophora extracellular calcium-sensing receptor-like 6
Stylophora extracellular calcium-sensing receptor-like 23
Stylophora extracellular calcium-sensing receptor-like 7
Stylophora extracellular calcium-sensing receptor-like 13
Stylophora extracellular calcium-sensing receptor-like 4
Stylophora extracellular calcium-sensing receptor-like 8
Stylophora extracellular calcium-sensing receptor-like 24
Stylophora extracellular calcium-sensing receptor-like 19
Stylophora extracellular calcium-sensing receptor-like 17
Stylophora extracellular calcium-sensing receptor-like 18
Stylophora extracellular calcium-sensing receptor-like 15
Stylophora extracellular calcium-sensing receptor-like 16
Stylophora extracellular calcium-sensing receptor-like 22
Stylophora extracellular calcium-sensing receptor-like 11
Stylophora extracellular calcium-sensing receptor-like 5
Stylophora extracellular calcium-sensing receptor-like 14
Stylophora extracellular calcium-sensing receptor-like 10
Stylophora extracellular calcium-sensing receptor-like 27
Stylophora extracellular calcium-sensing receptor-like 3
Stylophora extracellular calcium-sensing receptor-like 9
Homo sapiens taste receptor coreceptor TAS1R3
Homo sapiens taste receptor umami TAS1R1
Homo sapiens taste receptor sweet TAS1R2
Homo sapiens membrane androgen receptor GPRC6A
Homo sapiens extracellular calcium-sensing receptor isoform 2
Mus musculus vomeronasal receptor Vmn2r116
Mus musculus vomeronasal receptor Vmn2r26
Stylophora extracellular calcium-sensing receptor-like 1
Stylophora extracellular calcium-sensing receptor-like 2
Trichoplax putative calcium-sensing receptor 1
Octopus extracellular calcium-sensing receptor-like
Trichoplax putative calcium-sensing receptor 2
Trichoplax putative calcium-sensing receptor 3
Trichoplax calcium sensing receptor-like 2
Trichoplax calcium sensing receptor-like 3
Trichoplax calcium sensing receptor-like 1
Trichoplax calcium sensing receptor-like 29
Trichoplax calcium sensing receptor-like 27
Trichoplax calcium sensing receptor-like 25
Trichoplax calcium sensing receptor-like 26
Trichoplax calcium sensing receptor-like 28
Trichoplax calcium sensing receptor-like 5
Trichoplax calcium sensing receptor-like 10
Trichoplax calcium sensing receptor-like 11
Trichoplax calcium sensing receptor-like 12
Trichoplax calcium sensing receptor-like 4
Trichoplax calcium sensing receptor-like 14
Trichoplax calcium sensing receptor-like 13
Trichoplax calcium sensing receptor-like 23
Trichoplax calcium sensing receptor-like 24
Trichoplax calcium sensing receptor-like 22
Trichoplax calcium sensing receptor-like 19
Trichoplax calcium sensing receptor-like 18
Trichoplax calcium sensing receptor-like 17
Trichoplax calcium sensing receptor-like 16
Trichoplax calcium sensing receptor-like 15
Trichoplax calcium sensing receptor-like 20
Trichoplax calcium sensing receptor-like 21
Trichoplax calcium sensing receptor-like 7
Trichoplax calcium sensing receptor-like 6
Trichoplax calcium sensing receptor-like 8
Trichoplax calcium sensing receptor-like 9
Bilateria mGluR
Bilateria extracellular calcium-sensing, taste and vomeronasal
0.6
Placozoa
Porifera
Cnidaria
Bilateria

Supplemental figure S5. Maximum Likelihood phylogenetic tree of metabotropic GABA receptors. Numbers denote ultrafast bootstrap support near nodes. Sequences were aligned using E-INS-i algorithm implemented in MAFFT online. Alignment consisted of 5312 positions, 1376 of them parsimony-informative.

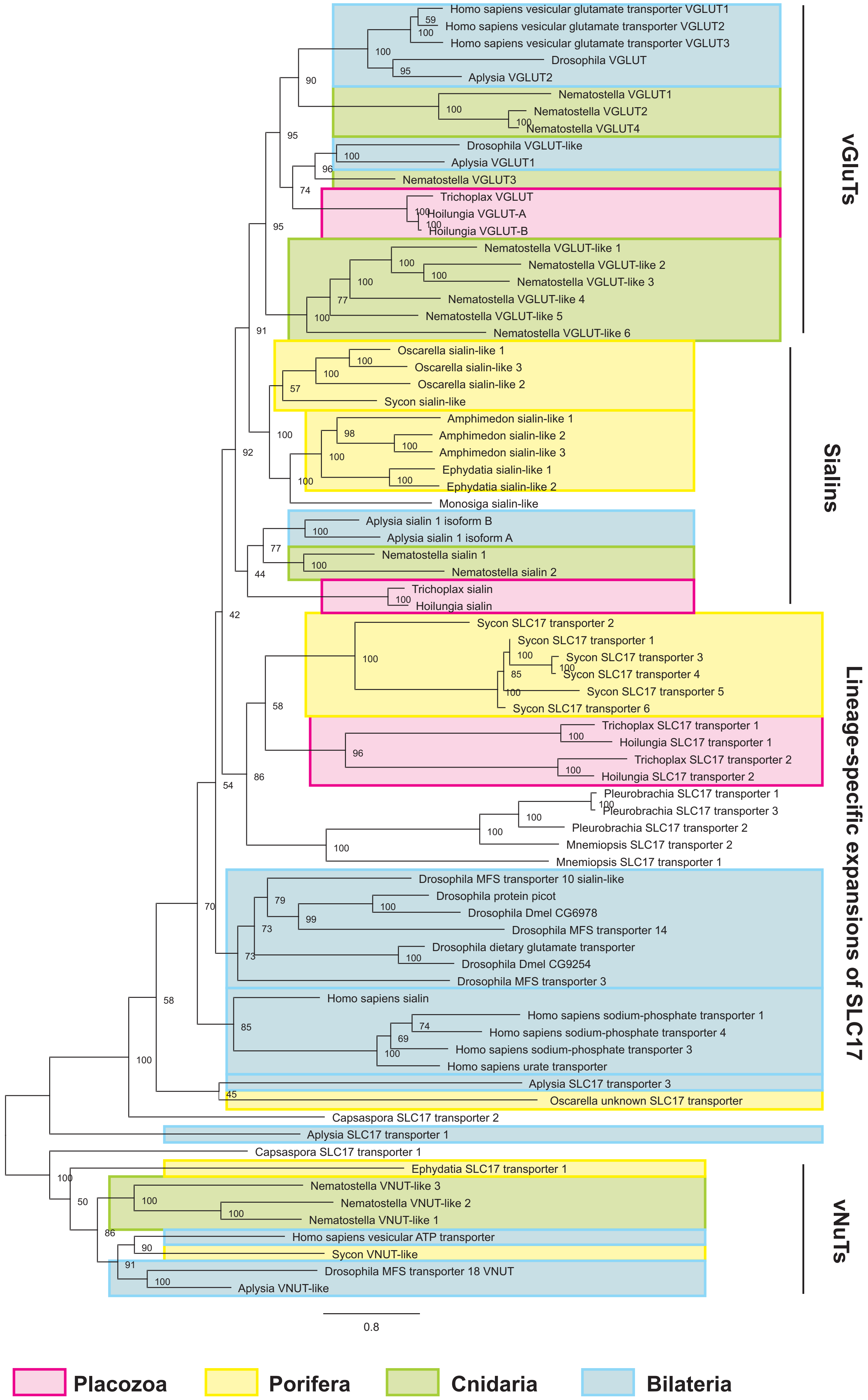
Homo sapiens vesicular glutamate transporter VGLUT1
Homo sapiens vesicular glutamate transporter VGLUT2
Homo sapiens vesicular glutamate transporter VGLUT3
Drosophila VGLUT
Aplysia VGLUT2
Nematostella VGLUT1
Nematostella VGLUT2
Nematostella VGLUT4
Drosophila VGLUT-like
Aplysia VGLUT1
Nematostella VGLUT3
Trichoplax VGLUT
Hoilungia VGLUT-A
Hoilungia VGLUT-B
Nematostella VGLUT-like 1
Nematostella VGLUT-like 2
Nematostella VGLUT-like 3
Nematostella VGLUT-like 4
Nematostella VGLUT-like 5
Nematostella VGLUT-like 6
Oscarella sialin-like 1
Oscarella sialin-like 3
Oscarella sialin-like 2
Sycon sialin-like
Amphimedon sialin-like 1
Amphimedon sialin-like 2
Amphimedon sialin-like 3
Ephydatia sialin-like 1
Ephydatia sialin-like 2
Monosiga sialin-like
Aplysia sialin 1 isoform B
Aplysia sialin 1 isoform A
Nematostella sialin 1
Nematostella sialin 2
Trichoplax sialin
Hoilungia sialin
Sycon SLC17 transporter 2
Sycon SLC17 transporter 1
Sycon SLC17 transporter 3
Sycon SLC17 transporter 4
Sycon SLC17 transporter 5
Sycon SLC17 transporter 6
Trichoplax SLC17 transporter 1
Hoilungia SLC17 transporter 1
Trichoplax SLC17 transporter 2
Hoilungia SLC17 transporter 2
Pleurobrachia SLC17 transporter 1
Pleurobrachia SLC17 transporter 3
Pleurobrachia SLC17 transporter 2
Mnemiopsis SLC17 transporter 2
Mnemiopsis SLC17 transporter 1
Drosophila MFS transporter 10 sialin-like
Drosophila protein picot
Drosophila Dmel CG6978
Drosophila MFS transporter 14
Drosophila dietary glutamate transporter
Drosophila Dmel CG9254
Drosophila MFS transporter 3
Homo sapiens sialin
Homo sapiens sodium-phosphate transporter 1
Homo sapiens sodium-phosphate transporter 4
Homo sapiens sodium-phosphate transporter 3
Homo sapiens urate transporter
Aplysia SLC17 transporter 3
Oscarella unknown SLC17 transporter
Capsaspora SLC17 transporter 2
Aplysia SLC17 transporter 1
Capsaspora SLC17 transporter 1
Ephydatia SLC17 transporter 1
Nematostella VNUT-like 3
Nematostella VNUT-like 2
Nematostella VNUT-like 1
Homo sapiens vesicular ATP transporter
Sycon VNUT-like
Drosophila MFS transporter 18 VNUT
Aplysia VNUT-like
vGluTs
Sialins
Lineage-specific expansions of SLC17
vNuTs
0.8
Placozoa
Porifera
Cnidaria
Bilateria

Supplemental figure S6. Bayesian phylogenetic tree of vesicular glutamate and related transporters (SLC17 family). Numbers denote bayesian posterior probability near nodes. Analysis was run for 10 million generations, and convergence was checked with Tracer. Sequences were aligned using E-INS-i algorithm implemented in MAFFT online. Alignment consisted of 1036 positions, 603 of them parsimony-informative.
